# The impact of hyperglycemia upon BeWo trophoblast cell metabolic function: A multi-OMICS and functional metabolic analysis

**DOI:** 10.1371/journal.pone.0283118

**Published:** 2023-03-17

**Authors:** Zachary J. W. Easton, Xian Luo, Liang Li, Timothy R. H. Regnault

**Affiliations:** 1 Department of Physiology and Pharmacology, Western University, London, Ontario, Canada; 2 The Metabolomics Innovation Centre, University of Alberta, Edmonton, Alberta, Canada; 3 Department of Chemistry, University of Alberta, Edmonton, Alberta, Canada; 4 Department of Obstetrics and Gynaecology, London Health Science Centre-Victoria Hospital, London, Ontario, Canada; 5 Children’s Health Research Institute, London, Ontario, Canada; 6 Lawson Health Research Institute, London, Ontario, Canada; University of Illinois, UNITED STATES

## Abstract

Pre-existing and gestationally-developed diabetes mellitus have been linked with impairments in placental villous trophoblast cell metabolic function, that are thought to underlie the development of metabolic diseases early in the lives of the exposed offspring. Previous research using placental cell lines and *ex vivo* trophoblast preparations have highlighted hyperglycemia is an important independent regulator of placental function. However, it is poorly understood if hyperglycemia directly influences aspects of placental metabolic function, including nutrient storage and mitochondrial respiration, that are altered in term diabetic placentae. The current study examined metabolic and mitochondrial function as well as nutrient storage in both undifferentiated cytotrophoblast and differentiated syncytiotrophoblast BeWo cells cultured under hyperglycemia conditions (25 mM glucose) for 72 hours to further characterize the direct impacts of placental hyperglycemic exposure. Hyperglycemic-exposed BeWo trophoblasts displayed increased glycogen and triglyceride nutrient stores, but real-time functional readouts of metabolic enzyme activity and mitochondrial respiratory activity were not altered. However, specific investigation into mitochondrial dynamics highlighted increased expression of markers associated with mitochondrial fission that could indicate high glucose-exposed trophoblasts are transitioning towards mitochondrial dysfunction. To further characterize the impacts of independent hyperglycemia, the current study subsequently utilized a multi-omics approach and evaluated the transcriptomic and metabolomic signatures of BeWo cytotrophoblasts. BeWo cytotrophoblasts exposed to hyperglycemia displayed increased mRNA expression of *ACSL1*, *HSD11B2*, *RPS6KA5*, *and LAP3* and reduced mRNA expression of *CYP2F1*, and *HK2*, concomitant with increased levels of: lactate, malonate, and riboflavin metabolites. These changes highlighted important underlying alterations to glucose, glutathione, fatty acid, and glucocorticoid metabolism in BeWo trophoblasts exposed to hyperglycemia. Overall, these results demonstrate that hyperglycemia is an important independent regulator of key areas of placental metabolism, nutrient storage, and mitochondrial function, and these data continue to expand our knowledge on mechanisms governing the development of placental dysfunction.

## Introduction

The rates of diabetes mellitus (DM) during pregnancy have increased substantially over the past several decades [1]. It is currently estimated that up to 1 in 10 pregnancies worldwide are impacted by maternal DM [2,3], however these rates may be even higher in certain at risk demographics including Indigenous populations [4]. These increases are particularly concerning as maternal DM during pregnancy, regardless of whether it is pre-existing (such as in type 1 DM or type 2 DM) or develops during gestation (gestational DM (GDM)), is associated with poor fetal health outcomes [5–8]. Specifically, children exposed to DM during intrauterine development have been found to be at a greater risk of developing non-communicable diseases such as obesity, metabolic syndrome, and impaired insulin sensitivity early in their lives [5,9–12]. Understanding the underlying mechanisms that link maternal DM during pregnancy to the development of non-communicable diseases in offspring is critical to develop appropriate prenatal clinical management practices that help reduce health risks to the next generations.

As the placenta is responsible for nutrient, gas and waste exchange between mother and fetus, specific functional alterations in this organ may be important in facilitating the intrauterine programming of metabolic disorders in DM-exposed offspring. Unsurprisingly, morphological and functional abnormalities have been found to be highly prevalent in placentae of diabetic pregnancies [13,14]. For example, diabetic placentae are often heavier [15–17], and display increased glycogen and triglyceride content [18–23], that is suggestive of altered nutrient storage and processing by the placenta and subsequently altered nutrient delivery to the developing fetus. This increase in nutrient storage in DM placentae has been thought to modulate trans-placental nutrient transport and fetal growth trajectories [24,25]. Additionally, the progenitor cytotrophoblasts (CT) and differentiated syncytiotrophoblasts (SCT) cells of the placenta villous trophoblast layer (cells that form the materno-fetal exchange barrier and are a primary site for placental energy (ATP) production) have been found to have impaired mitochondrial function in response to maternal DM that may further impact placental nutrient handling [26]. In particular, cultured primary CT and SCT cells from GDM pregnancies have been found to have reduced basal and maximal mitochondrial respiratory (oxidative) activity compared to non-diabetic control trophoblasts [27,28]. Additionally, pre-existing DM has been found to impact the activities of individual placental Electron Transport Chain (ETC) complexes in whole placental lysates, highlighted by reduced complex I, II and III activity in type 1 DM placentae, and reduced complex II and III activity in type 2 DM placentae [29]. Overall, these studies suggest that impaired placental nutrient storage and mitochondrial oxidative function may be implicated in the development of metabolic diseases in DM-exposed offspring.

Previous work with placental cell lines and *ex vivo* placental explant preparations have demonstrated that hyperglycemia (a hallmark symptom of both pre-existing and gestationally-developed DM) is an important regulator of placental metabolic function. For example, explants from uncomplicated pregnancies were found to have altered lipid processing when cultured under hyperglycemic (HG) conditions (25 mM glucose) for 18 hours [22]. Further reports have highlighted transcriptomic and metabolomic markers indicative of altered lipid metabolism, β-oxidation, and glycolysis functions in undifferentiated BeWo CT cells cultured under HG-conditions (25 mM) for 48 hours [30]. Independent hyperglycemia (30 mM glucose for 72h) has also been linked to increased Reactive Oxygen Species (ROS) generation in undifferentiated BeWo CTs [31], which may directly promote the development mitochondrial oxidative damage [32,33]. Overall, these reports have suggested that hyperglycemia may independently facilitate the development of aberrant placental metabolic function in diabetic pregnancies. However, the direct and independent impacts of hyperglycemia on placental mitochondrial respiratory (oxidative) function are poorly understood. Previously, elevated glucose levels (25 mM for 48h) have also been associated with altered mitochondrial activity in BeWo CT cells when assessed by endpoint tetrazolium salt (MTT) assay [34]. However, an interrogation of mitochondrial respiratory activity of HG-exposed trophoblast cells using recently developed real-time functional readouts (such as the Seahorse XF Analyzer), as has been performed with DM-exposed Primary Human Trophoblasts (PHT), [27–29] is warranted. In addition, the direct impacts of hyperglycemia on glycogen and lipid nutrient stores of placental trophoblasts and the underlying mechanisms governing placental nutrient storage in HG-conditions remains ill defined.

The first objective of the current study was to characterize the impacts of independent hyperglycemia on placental mitochondrial respiratory activity and nutrient storage by evaluating both undifferentiated BeWo CTs and differentiated BeWo SCTs following a relatively prolonged 72-hour HG (25 mM) exposure. Recently, the integration of transcriptomics with metabolomics has been identified as a useful method to elucidate cellular mechanisms that underlie pathological placental development in pre-clinical models [30,35,36]. In turn, the second objective of this study was to utilize a multi-omics research approach to thoroughly characterize potential mechanisms leading to altered metabolic function in high-glucose exposed BeWo progenitor CT cells. It was postulated that HG-culture conditions would be associated with increased nutrient storage and impaired mitochondrial respiratory function in BeWo CT and SCT cells, in association with altered transcriptome and metabolome signatures in BeWo CT cells indicative of altered metabolic function.

## Materials and methods

### Materials

All materials were purchased from Millipore Sigma (Oakville, Canada) unless otherwise specified.

### Cell culture conditions

BeWo (CCL-98) trophoblast cells were purchased from the American Type Culture Collection (ATCC; Cedarlane Labs, Burlington, Canada). Cells were cultured in F12K media (Gibco, ThermoFisher Scientific, Mississauga, Canada) as recommended by the ATCC, and supplemented with 10% Fetal Bovine Serum (Gibco) and 1% Penicillin-Streptomycin (Invitrogen, ThermoFisher Scientific, Mississauga, Canada). All cells were utilized between passages 5–15 and were maintained at 37°C and 5% CO_2_/95% atmospheric air.

**Fig 1:**
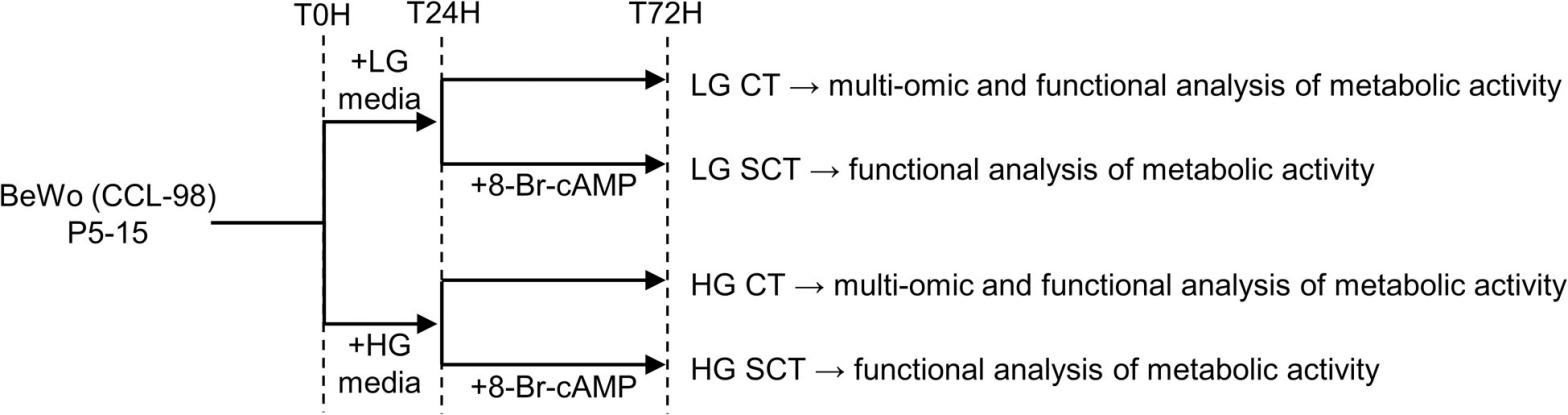
Schematic of 72-hour HG cell culture protocol. BeWo trophoblasts were plated in F12K media and allowed to adhere to culture plates overnight. At T0H cells were treated with low glucose (LG, 7 mM) or hyperglycemic (HG, 25 mM) supplemented F12K media. At T24H subsets of BeWo trophoblasts were treated with 250 μM 8-Br-cAMP to induce CT-to-SCT differentiation. Cell media was replenished every 24 hours and cells were collected at T72H for analysis of metabolic function.

The F12K media contained 7 mM of glucose, a relatively physiological glucose level, and was utilized for low-glucose (LG) controls. F12K media was supplemented to 25 mM glucose for hyperglycemic (HG) culture treatments as previously utilized with BeWo trophoblasts [30,34]. BeWo trophoblasts cells were plated in LG F12K media at the specifically stated experimental densities and allowed to adhere to cell culture plates overnight before being treated with HG culture media. Cell media was replenished every 24 hours. At T24h and T48h subsets of BeWo trophoblasts were treated with 250 μM 8-Br-cAMP to induce differentiation from cytotrophoblast-like (CT) cells to SCT cells. Cell cultures were collected after 72 hours of high glucose exposure. A schematic of the HG culture protocol is available in **Fig 1**.

### Cell viability of HG-cultured BeWo trophoblasts

BeWo trophoblasts were plated at 7.5x10^3^ cells/well in black walled 96-well cell culture plates and cultured as described. At T72h cell viability of both CT and SCT cultures was assessed via the CellTiter-Fluor cell viability assay (Promega Corporation, Madison WI, USA) as per manufacturer’s instructions.

### Analysis of BeWo cell fusion under HG culture conditions

To determine the potential of HG culture conditions to impact the ability of BeWo trophoblasts to differentiate into SCT cells, cell fusion of 8-Br-cAMP stimulated cells (expressed as percent loss of the tight junction protein zona occludens-1 (ZO-1)) was examined. In brief, BeWo cells were plated at 1.4x10^5^ cells/well in 6-well plates containing coverslips coated with laminin (2 μg/cm^2^) and grown under HG conditions as described. Cellular expression of ZO-1 was then examined via immunofluorescent microscopy as previously detailed [37].

### RT-qPCR analysis of BeWo syncytialization under HG conditions

The expression of the transcription factor Ovo Like Transcriptional Repressor 1 (OVOL1) as well as human chorionic gonadotropin subunit beta (CGB) was additionally analyzed to ensure cell fusion in 8-Br-cAMP stimulated BeWo cells was associated with increased expression of syncytialization-related genes. In brief, BeWo trophoblasts were plated at 3.5x10^5^ cells/plate in 60mm cell culture plates and cultured as described above. At T72h cells were collected in TRIzol reagent (Invitrogen), and total RNA was extracted as per the manufacturer’s protocol. RNA integrity was assessed via formaldehyde gel electrophoresis, and RNA concentration was quantified by Nanodrop Spectrophotometer 2000 (NanoDrop Technologies, Inc., Wilmington, DE, USA). RNA (2 μg) was then reverse transcribed with the High-Capacity cDNA Reverse Transcription Kit (Applied Biosystems; ThermoFisher Scientific). RT-qPCR was then performed via the CFX384 Real Time system (Bio-Rad, Mississauga, Canada). Relative gene expression of *OVOL1* [38] and *CGB* [39] was then determined using the ΔΔCt method with the geometric mean of *PSMB6* and *ACTB* utilized as a reference gene. Primer sequences and their efficiencies are available in **S1 Table in S2 File**.

### Seahorse XF Mito Stress Test quantification of mitochondrial respiratory activity

BeWo cells were plated at 7.5x10^3^ cells/well in Seahorse XF24 V7PS plates and cultured under LG and HG conditions as described above. At T72h mitochondrial activity was assessed using the Seahorse XF Mito Stress Test, as previously optimized for BeWo trophoblast cells [37]. In brief, oxygen consumption rate (OCR) of cell culture media was quantified as a proxy measure of mitochondrial respiratory activity. Subsequent injections of oligomycin (1.5 μg/mL), dinitrophenol (50 μM) and Rotenone and Antimycin A (0.5 μM each) were used to interrogate the basal respiration, maximal respiration, proton leak, spare respiratory capacity and coupling efficiency parameters of mitochondrial respiratory activity. OCR measures were normalized to cellular DNA content using Hoechst dye fluorescence as previously described [37].

### Seahorse XF Glycolysis Stress Test quantification of BeWo trophoblast glycolytic activity

BeWo cells were plated at 7.5x10^3^ cells/well in Seahorse XF24 V7PS plates and cultured under LG and HG conditions as described above. At T72h glycolytic activity was assessed via the Seahorse XF Glycolysis Stress Test, as previously optimized for BeWo trophoblasts [37]. In brief, Extracellular Acidification Rate (ECAR) of cell culture media was assessed to quantify glycolytic activity in BeWo trophoblasts. Injections of glucose (10 mM), oligomycin (1.5 μg/mL) and 2-deoxyglucose (50 mM) were utilized to interrogate the basal glycolytic rate, maximal glycolytic rate, glycolytic reserve, and non-glycolytic acidification parameters of glycolytic function. ECAR data was normalized to cellular DNA content using Hoechst dye fluorescence as previously described [37].

### Immunoblot analysis of protein abundance

BeWo trophoblasts were plated at 9.5x10^5^ cells/plate in 100 mm cell culture dishes and cultured under LG and HG conditions as described. At T72h cells were washed once in ice-cold PBS and subsequently lysed in radioimmunoprecipitation assay (RIPA) buffer supplemented with protease and phosphatase inhibitors as previously described [37]. Protein concentrations were then adjusted to 2 μg/μL in Laemmli loading buffer (62.5 mM Tris-Cl (pH 6.8); 2% SDS 10% glycerol; 0.002% bromophenol blue; 4% β-mercaptoethanol).

The relative abundance of target proteins was then determined via SDS-PAGE gel electrophoresis. In brief, protein lysates were separated on acrylamide gels (10–15%), and separated proteins were transfer to polyvinylidene fluoride (PVDF) membranes (EMD Millipore, Fisher Scientific). Total lane protein was then determined via Ponceau stain (0.1% Ponceau-S in 5% acetic acid) and utilized to normalize densitometry values. PVDF membranes were blocked in 5% dry-milk protein or 5% Bovine Serum Albumin (BSA, BioShop Canada Inc., Burlington, Canada) and membranes were incubated overnight at 4°C with respective antibody solutions (**S2 Table in S2 File**). Membranes were then washed 3 times with Tris-buffered Saline with 0.1% Tween-20 (TBST) and incubated with respective secondary antibodies for 1 hour at room temperature (**S2 Table in S2 File**). Membranes were washed 3 more times in TBST and imaged on a ChemiDoc Imager (Bio-Rad) using Clarity western ECL substrate (Bio-Rad). Protein band abundance and total lane protein (ponceau) were quantified with Image-Lab Software (Bio-Rad).

### Analysis of ETC complex I and II activity in HG BeWo trophoblasts

BeWo trophoblasts were plated at 3.5x10^5^ cells/plate in 60mm cell culture dishes and cultured under LG and HG conditions as described. At T72h cells were washed once with PBS and detached from cell culture plates by scraping. Cells were then pelleted (400g, 5 mins), snap frozen in liquid nitrogen and stored at -80°C until analyzed. Cell pellets were subsequently lysed and Complex I activity was assessed as the rate of rotenone-sensitive NADH oxidation, and Complex II activity was assessed as the rate of DCPIP oxidation as previously detailed [37]. ETC complex activity assays were normalized to cell lysate protein content via Bicinchoninic Acid (BCA) assay (Pierce, ThermoFisher Scientific) as per manufacturer’s instructions.

### Analysis of metabolic enzyme activities in HG BeWo trophoblasts

BeWo trophoblasts were plated at 3.5x10^5^ cells/plate in 60mm cell culture dishes and cultured under LG and HG conditions as detailed above. To examine the enzyme activities of Lactate Dehydrogenase (LDH) and Citrate Synthase (CS), cells were collected by scraping and lysed in glycerol lysis buffer (20 mM Na_2_HPO_4_, 0.5 mM EDTA, 0.1% Triton X-100, 0.2% BSA, 50% glycerol) containing protease and phosphatase inhibitors as previously described [37,40]. LDH activity was assessed as the rate of NADH oxidation, and CS activity was assessed as the rate of Ellman’s reagent consumption as previously detailed [37]. The enzyme activity of LDH and CS were normalized to cell lysate protein content via BCA assay.

Additional BeWo cultures were collected to analyze the activity of the E1 (rate-limiting) subunit of the Pyruvate Dehydrogenase (PDH) complex. PDH-E1 subunit was assessed on freshly collected BeWo cells as the rate of DCPIP oxidation and normalized to protein content via BCA assay as previously detailed [37].

### Nutrient storage in HG BeWo trophoblasts

BeWo trophoblasts were plated at 3.5x10^5^ cells/plate in 60mm cell culture dishes and cultured under LG and HG conditions. At T72h cells were washed with PBS and collected into fresh PBS (1.5 mL) by scrapping. Cells were then pelleted (400g, 5 minutes) and the PBS was aspirated.

To determine cellular glycogen content, the cell pellets were lysed in 200 μL ddH_2_O and samples were boiled for 10 minutes to inactivate cell enzymes. Samples were stored at -20 until glycogen content was analyzed. Samples were diluted 5-fold and glycogen content was analyzed via the Glycogen Assay Kit (ABCAM, ab65620) as per manufacturer’s protocol. Glycogen content was then normalized to cell lysate protein content via BCA assay.

To determine cellular triglyceride accumulation, the collected cell pellets were snap frozen in liquid nitrogen and stored at -80°C until analyzed. Cells were then lysed, and cellular triglyceride content was analyzed via the Triglyceride Assay Kit (ABCAM, ab178780) as per kit instructions. Triglyceride content was normalized to cell lysate protein content determined via BCA assay.

### Transcriptomic analysis of gene expression changes in HG BeWo CT cells

BeWo CT cells were plated at 3.5x10^5^ cells/plate in 60 mm cell culture dishes and grown under LG and HG culture conditions as described. At T72h cells with washed once with PBS and collected in 900 μL TRIzol Reagent and stored at -80°C. Samples were then shipped to the Genome Québec Innovation Centre for transcriptomic analysis via Clariom S mRNA microarray. Automated RNA extraction was completed via QIAcube Connect (Qiagen, Toronto, Canada). RNA content was then quantified via NanoDrop Spectrophotometer 2000 (Nanodrop Technologies, Inc) and RNA integrity was determined by Bioanalyzer 2100 (Agilent Technologies, Waldbronn, Germany). All extracted RNA samples had an RNA Integrity Number (RIN) greater than 9.5. RNA was then processed via the Affymetrix Whole Transcript 2 workflow and analyzed using a Clariom S human mRNA microarray. In brief, sense-stranded cDNA was synthesized from 100 ng total RNA, and subsequently fragmented, and labelled with the GeneChip WT Terminal Labeling Kit (ThermoFisher Scientific) as per manufacturer’s instructions. Labelled DNA was then hybridized to Clariom S human GeneChips (ThermoFisher Scientific) and incubated at 45°C in the GeneChip Hybridization oven 640 (Affymetrix, ThermoFisher Scientific) for 17 hours at 60 rpm. GeneChips were washed using GeneChip Hybridization Wash and Stain Kit (ThermoFisher Scientific) according to manufacturer’s specifications. Microarray chips were scanned on a GeneChip scanner 3000 (ThermoFisher Scientific).

Microarray data was then analyzed via Transcriptome Analysis Console v4.0 (ThermoFisher Scientific), and raw data was normalized using the Robust Multiple-Array Averaging (RMA) method. HG-treated samples were paired with respective LG-control for each cell collection for analysis. Genes with a ≥ ±1.3 fold-change (FC) vs LG-control and raw-p < 0.05 were determined to be differentially expressed.

RMA-normalized microarray signals (for all identified genes) were then imported into the Gene Set Enrichment Analysis (GSEA) v4.2.3 software (Broad Institute Inc., Cambridge MA, USA) [41,42], and the genes annotated on the microarray chip were ranked via a signal-to-noise score. The ranked gene list was subsequently uploaded to the Web-Based Gene Set Analysis Toolkit (WebGestalt) to conduct GSEA analysis using the KEGG, Wikipathways and Reactome functional gene sets, as well as the Gene Ontology (GO) biological processes and molecular function gene sets [43]. Identified pathways with a False Discovery Rate-corrected p-value <0.25 were determined to be significantly enriched the HG or LG-cultured BeWo CT cells as has previously been reported [42,44].

### RT-qPCR validation of differentially expressed genes identified by mRNA microarray

RT-qPCR was utilized to validate differentially expressed genes involved in metabolic pathways that were highlighted by the mRNA microarray. The RNA utilized for the microarray was returned by Genome Québec and 2 μg was reverse transcribed as described above. The CT samples previously utilized to examine expression of syncytialization-related genes were additionally utilized to validate the differentially expressed genes identified in the microarray. RT-qPCR was then performed and analyzed using the ΔΔCt with the geometric mean of *ACTB* and *PSMB6* used as a reference. Primer sequences of validated targets and their efficiencies are available in **S1 Table in S1 File**.

### Untargeted metabolomic profiling of HG-treated BeWo CT cells

BeWo trophoblasts were plated at a density of 2x10^6^ cells/plate in 150mm cell culture plates and cultured under LG and HG-conditions as described. At T72h cell media was aspirated and the cells were washed three times with cold PBS. Pre-cooled methanol (-20°C) was then added to quench cellular metabolic processes. Cells were then scraped and collected into microcentrifuge tubes, and the methanol was evaporated with a gentle flow of nitrogen gas. Samples were then frozen at -80°C, and subsequently were lyophilized to remove any residual moisture. The samples were then sent to The Metabolomics Innovation Centre (Edmonton, Canada) for subsequent metabolomics analysis via a High-Performance Chemical Isotope Labelling (HP CIL) liquid chromatography mass spectrometry (LC-MS) approach [45,46].

Samples were reconstituted in 50% methanol and freeze-thaw cycles were utilized to lyse the cells. The lysed samples were centrifuged at 16000 g at 4°C and the supernatants were transferred to new vials. The supernatants were dried down and re-dissolved in 41 μL of water. The total concentrations of metabolite were then determined via the NovaMT Sample Normalization kit (Edmonton, Alberta). Water was added to adjust all the concentrations of samples to 2 mM. The samples were split into five aliquots for respective labeling methods. Each of the individual samples was labeled by ^12^C-DnsCl, base activated ^12^C-DnsCl, ^12^C-DmPA Br, and ^12^C-DnsHz, for amine-/phenol-, hydroxyl-, carboxyl, and carbonyl- metabolomic profiling, respectively [47]. A pooled sample was generated by mixing each individual sample and was labeled by ^13^C-reagent, accordingly. Each ^12^C-labeled individual sample was then mixed with a ^13^C- labeled reference sample by equal volume, and the mixtures were injected onto LC-MS for analysis. The LC-MS system was the Agilent 1290 LC (Agilent Technologies) linked to the Bruker Impact II QTOF Mass Spectrometer (Bruker Corporation, Billerica, US). LC-MS data was then exported to.csv files with Bruker DataAnalysis 4.4 (Bruker Corporation), the exported data were then uploaded to IsoMS pro v1.2.7 for data quality check and data processing.

Metabolite peak pairs were then identified using a three-tier approach [47]. In tier 1, peak pairs were identified by searching against a labelled metabolite library (CIL library) based on accurate mass and retention time. In tier 2, the remaining peak pairs were matched by searching against a linked identity library (LI library), containing predicted retention time and accurate mass information. In tier 3, the rest of peak pairs were matched by searching against MyCompoundID (MCID) library, containing accurate mass information of metabolites and their predicted products. Metabolites with ± 1.5 FC and False Discovery Rate raw-p<0.05 vs LG CT were determined to be differentially abundant in the HG-cultured BeWo CT cells.

Metabolites from tiers 1 and 2 (high confidence identifications) with an associated KEGG library numbers were subsequently identified for pathway analysis to elucidate the biological impacts of the differentially abundant metabolites. Enrichment in the Homo sapiens KEGG library and Small Molecule Pathway Database (SMPDB) was performed using MetaboAnalyst v5.0 (https://www.metaboanalyst.ca/). KEGG and SMPDB pathways with an FDR p<0.05 were determined to be significantly enriched.

### Integration of transcriptome and metabolome profiles

Differentially expressed genes (±1.3 FC, raw-p<0.05), and differentially abundant metabolites (±1.5 FC, raw-p<0.05) with a KEGG library identification library were then imported into the Joint Pathway Analysis Tool in MetaboAnalyst v5.0 to identify enriched pathways containing both altered genes and metabolites in the HG-cultured BeWo CT cells. Pathways with a raw-p<0.05 were determined to be significantly enriched.

### Statistical analysis of non-omics data

Data collected as a percentage (percent loss of ZO-1 staining data, spare respiratory capacity, coupling efficiency, and glycolytic reserve) were log-transformed and analyzed via Two-Way ANOVA (2WA) and Bonferroni’s Multiple Comparisons post-hoc test. A Randomized Block Design 2WA and Sidak’s Multiple Comparisons Test was utilized to analyze relative transcript abundances; relative protein abundances; metabolic activity parameters; and nutrient storage data, using raw data with data from each experimental replicate blocked together, as previously described [48]. These data were then expressed as percent of LG CT control for visualization in figures. Data from phosphorylated western blot targets were normalized to the average LG CT expression on each independent membrane prior to calculation of the phosphorylated protein-to-total protein abundance ratio, and subsequently analyzed via randomized block-design ANOVA, and Sidak’s multiple comparisons test. A paired T-test was utilized to analyze gene FC data between HG and LG BeWo CT cells in the RT-qPCR validation of the microarray. All data was analyzed for statistically significant outliers using the Rout method (Q = 1%). Statistical analysis was performed with GraphPad Prism 8 Software (GraphPad Software, San Diego, CA, USA).

## Results

### Characterization of BeWo viability and differentiation under high glucose culture conditions

**Fig 2:**
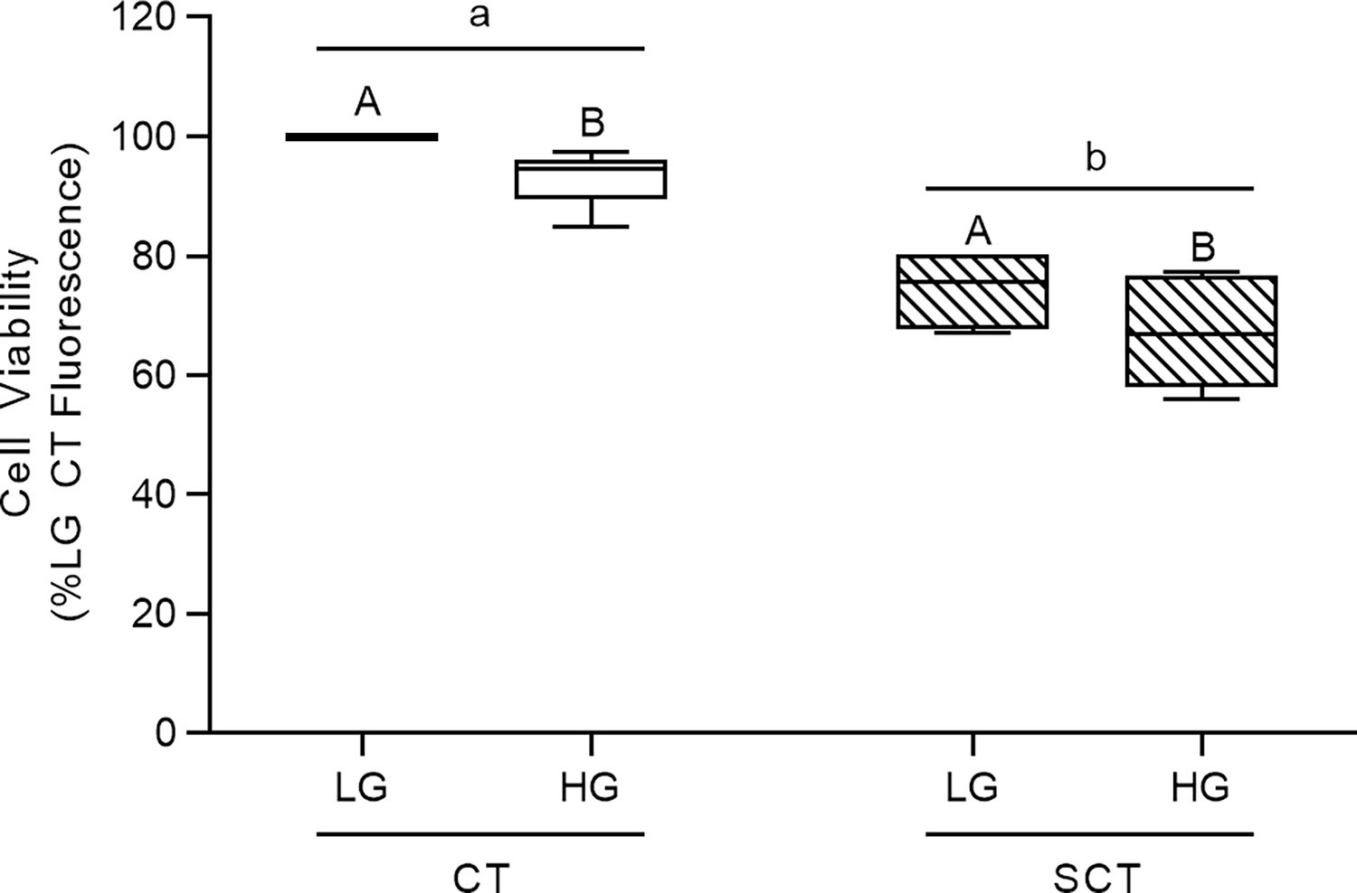
Viability of BeWo trophoblasts cultured under HG conditions for 72h. BeWo trophoblasts were cultured for 72H under HG culture conditions as described, and cell viability was assessed via the CellTiter-Flour cell viability assay. Data is presented as percent of LG CT cell viability (n = 6/group). Raw cell viability fluorescence data was analyzed via Two-Way Randomized block design ANOVA (2WA). Different lower-case letters denote differentiation state-dependent differences in viability between CT and SCT cultures, and different upper-case letter denote hyperglycemia-dependent differences in viability within each differentiation state (p<0.05).

HG culture conditions were associated with a mean 7% and 10% reduction in cell viability in BeWo CT and SCT cells respectively (**Fig 2;** p<0.01; n = 6/group). It is important to note that these viability data were consistent with previously reported values in BeWo trophoblasts and PHTs cultured under hyperglycemia (25 mM glucose) for 48h [30,49]. This highlights that a 72H HG-culture protocol does not impact the stability of cultured BeWo trophoblasts cells. Additionally, BeWo SCT cells displayed lower viability relative to BeWo CT cells consistent with trophoblast cells becoming less proliferative while undergoing syncytialization (**Fig 2;** p<0.001).

**Fig 3:**
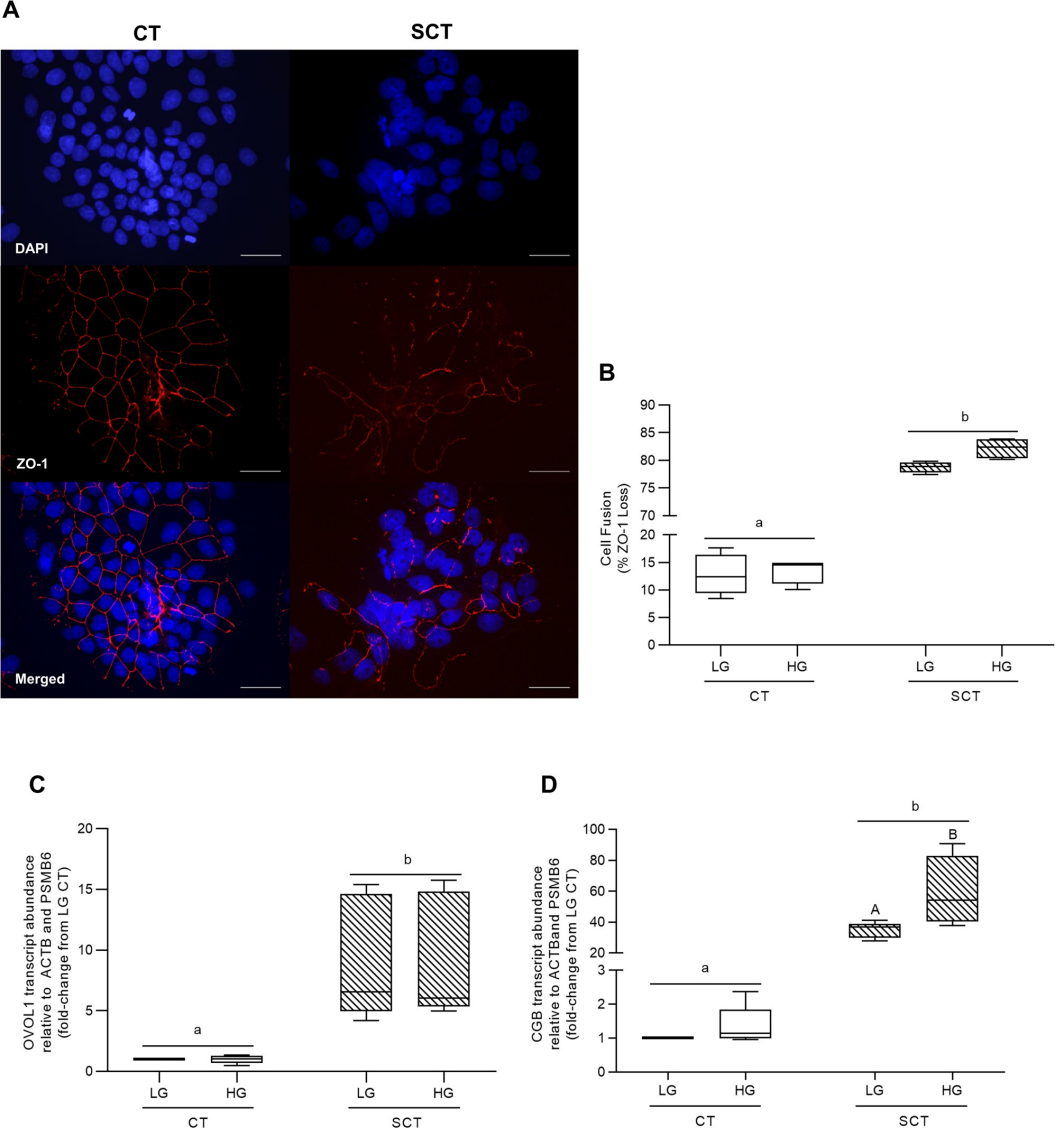
HG culture conditions do not impact BeWo SCT cell fusion but are associated with increased CGB transcript abundance at 72h. **(A)** Representative DAPI (blue); Zona occludens-1 (ZO1; red) and merged immunofluorescent images (scale bar = 50 μm) of BeWo CT and SCT cells. **(B)** Percent fusion of HG-cultured BeWo trophoblasts. Percent fusion data was log-transformed and analyzed via Two-Way Anova (2WA); data is expressed as the percentage of cells lacking ZO-1 expression (n = 4/group). BeWo cell syncytialization was additionally analyzed via quantifying mRNA transcript abundance of the syncytialization markers **(C)** OVOL1 and **(D)** CGB. Transcript abundance data was analyzed via Randomized Block 2WA and Sidak’s multiple comparisons test; data is present as transcript FC versus LG CT cultures (n = 5/group). Different lower-case letters denote differentiation state-dependent differences between CT and SCT cultures, and different upper-case letter denote hyperglycemia-dependent differences within each differentiation state (p<0.05).

BeWo SCT cultures displayed a greater loss of ZO-1 protein expression (and thus increased cell fusion) compared to BeWo CT cultures (**Fig 3A and 3B**; p<0.0001, n = 4/group). However, HG-culture conditions had no impact on ZO-1 protein expression in BeWo trophoblast cells (**Fig 3A and 3B**). BeWo SCT cultures additionally displayed increased relative transcript abundance of the syncytialization-associated transcription factor OVOL1 (**Fig 3C**; p<0.01, n = 5/group) as well as the syncytialization-associated hormone CGB (**Fig 3D**; p<0.01, n = 5/group). HG culture conditions in BeWo SCT cells was additionally associated with an increased transcript abundance of CGB compared to LG BeWo SCT cells (**Fig 3D**; p<0.05; 2WA: Interaction p<0.05).

### BeWo mitochondrial respiratory and glycolytic activity

High glucose culture conditions did not impact any of the parameters of mitochondrial respiratory function as assessed by the Seahorse XF Mito Stress Test (**Table 1;** n = 5/group). However, BeWo syncytialization was associated with reduced Spare Respiratory Capacity (**Table 1**; p<0.0001) and reduced Coupling Efficiency (**Table 1**; p<0.01) independent of culture glucose level.

**Table 1:** HG culture conditions do not impact BeWo trophoblast glycolytic activity.

Differentiation state	Treatment	Basal glycolytic rate (ECAR/μg DNA)	Max glycolytic rate (ECAR/μg DNA)	Reserve glycolytic capacity (% basal glycolysis)	Non-glycolytic acidification (ECAR/μg DNA)
CT	LG	75.71 ± 15.38	101.4 ± 20.92	133.8 ± 1.14	19.26 ± 4.43
	HG	67.54 ± 7.30	88.23 ± 11.82	129.5 ± 4.08	18.38 ± 3.10
SCT	LG	69.21 ± 12.67	84.45 ± 14.55	123.5 ± 3.70	20.50 ± 3.39
	HG	77.38 ± 6.78	88.01 ± 8.17	113.6 ± 1.45	23.30 ± 2.45
*Differentiation state difference		NS	NS	*	NS

Data are mean ± SEM (n = 5/group). Differences in mitochondrial respiratory activity parameters between observed BeWo CT and SCT differentiation states found in the two-way ANOVA test are denoted as

* p<0.05.

The parameters of glycolytic function as measured with the Seahorse XF Glycolysis Stress Test were not impacted by BeWo syncytialization, or by HG culture conditions (**Table 2**). Representative tracings from the Seahorse Mito Stress Test and Glycolysis Stress Test are available in **S1 Fig in S1 File**.

**Table 2:** Syncytialization but not HG culture conditions impacts BeWo trophoblast mitochondrial respiratory activity.

Differentiation state	Treatment	Basal respiration (OCR/μg DNA)	Maximal respiration (OCR/μg DNA)	Proton leak (OCR/μg DNA)	Spare respiratory capacity (% basal OCR)	Coupling efficiency (% basal OCR)
CT	LG	199.70 ± 39.08	249.10 ± 43.78	55.48 ± 8.45	129.00 ± 5.30	70.38 ± 3.34
	HG	173.40 ± 24.69	221.30 ± 36.78	46.72 ± 3.76	125.50 ± 6.79	70.58 ± 4.59
SCT	LG	173.00 ± 7.96	154.40 ± 11.97	78.20 ± 7.38	89.02 ± 5.68	55.04 ± 2.99
	HG	177.5 ± 19.07	168.30 ± 18.27	75.88 ± 12.12	95.76 ± 5.83	57.30 ± 3.80
*Differentiation state difference		NS	NS	NS	****	**

Data are mean ± SEM (n = 5/group). Differences in mitochondrial respiratory activity parameters between observed BeWo CT and SCT differentiation states found in the two-way ANOVA test are denoted as

** p<0.01

****p<0.0001.

### The impact of high glucose and syncytialization upon BeWo ETC complex protein abundance and activity

**Fig 4:**
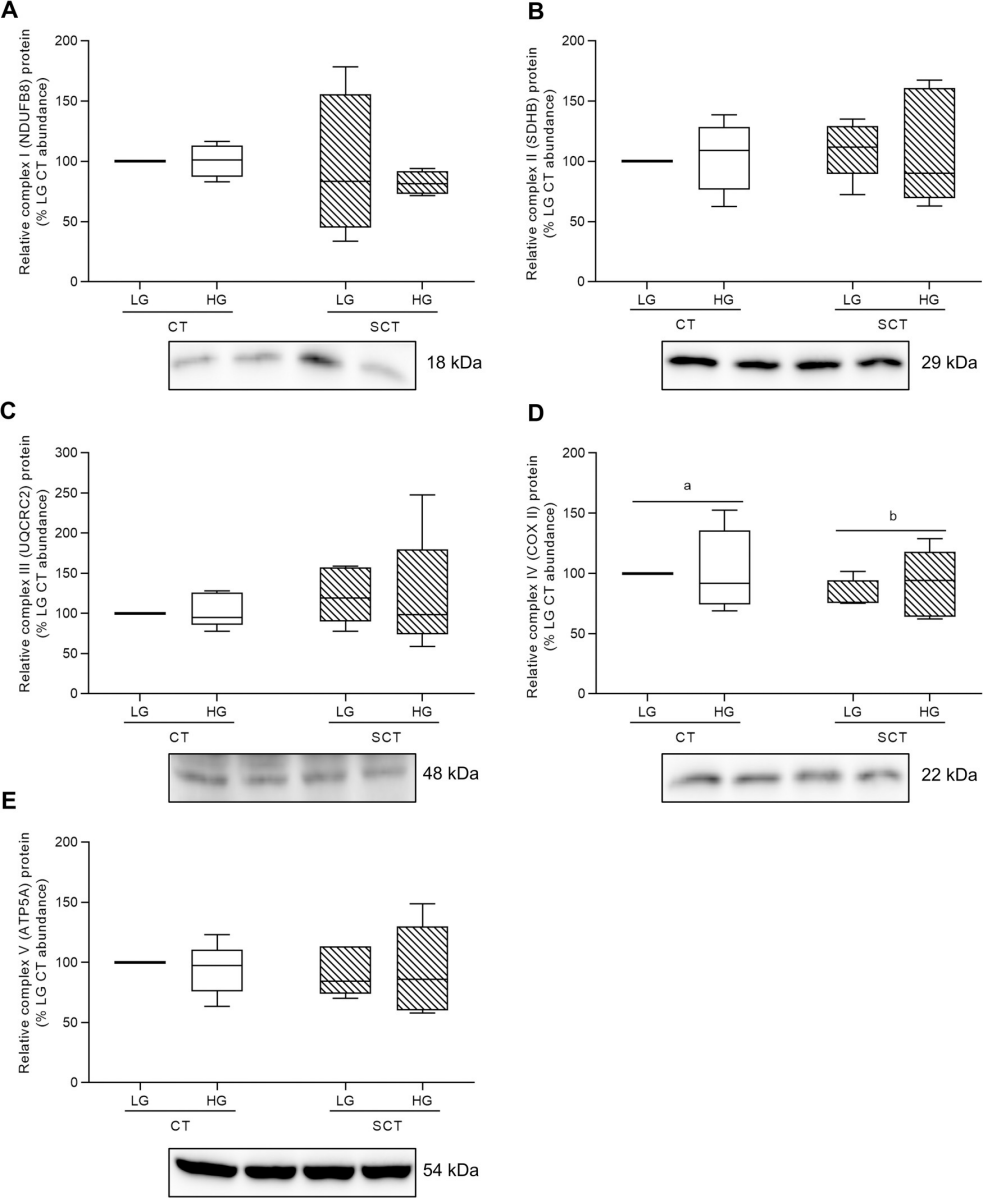
HG culture conditions do not impact protein expression of electron transport chain complexes in BeWo trophoblasts. Relative protein abundance of **(A)** Complex I (NDUFB8 subunit) **(B)** Complex II (SDHB subunit) **(C)** Complex III (UQCRC2 subunit) **(D)** Complex IV (COX II subunit), and **(E)** Complex V (ATP5A subunit) of the electron transport chain (ETC) in HG-cultured BeWo trophoblasts. Different lower-case letters denote differentiation state-dependent differences in ETC complex protein abundance between CT and SCT cells (n = 4-5/group; Two-Way Randomized Block ANOVA (2WA)). ETC complex protein band density was normalized to total lane protein (ponceau) for statistical analysis and the data is presented as percent of LG CT protein abundance for visualization. Full length representative images of ETC complex bands and ponceau staining of total lane protein are available in **S2 Fig in S1 File**.

High glucose levels did not impact protein abundance of ETC complex subunits in BeWo trophoblasts (**Fig 4A–4E**; n = 4-5/group). Likewise, HG-culture conditions did not affect ETC complex I or II activity in BeWo trophoblasts (**Table 3,** n = 5-6/group). However, BeWo syncytialization was associated with reduced ETC complex IV Cytochrome c oxidase subunit II (COXII) relative protein abundance in both LG and HG cultures (**Fig 4D**; p<0.05). Additionally, BeWo SCT cells displayed reduced activity of ETC complex II compared to CT cells independent of culture glucose level (**Table 3**; p<0.001, n = 6/group).

**Table 3:** Syncytialization but not HG-culture media impacts BeWo metabolic enzyme activity and individual ETC complex activity.

Differentiation state	Treatment	Citrate synthase	ETC complex I	ETC complex II	Lactate dehydrogenase	PDH-E1 subunit
		(U/mg protein)				
CT	LG HG	0.0126 ± 0.0034 0.0134 ± 0.0033	0.0348 ± 0.0049 0.0395 ± 0.0059	0.0470 ± 0.0036 0.0448 ± 0.0039	2.821 ± 0.277 3.095 ± 0.391	0.0046 ± 0.0006 0.0041 ± 0.0005
SCT	LG HG	0.0080 ± 0.0020 0.0070 ± 0.0015	0.0545 ± 0.0134 0.0512 ± 0.0110	0.0321 ± 0.0035 0.0330 ± 0.0039	3.589 ± 0.312 3.504 ± 0.359	0.0090 ± 0.0012 0.0098 ± 0.0014
*Differentiation state difference		NS	NS	***	NS	**

Data are mean ± SEM (n = 5-6/group). Differences in mitochondrial respiratory activity parameters observed between BeWo CT and SCT differentiation states found in the two-way ANOVA test are denoted as

** p<0.01

***p<0.001.

### Metabolic enzyme activity in HG-cultured BeWo trophoblasts

BeWo SCT cells displayed increased activity of the PDH-E1 subunit compared to BeWo CT cells regardless of glucose level (**Table 3;** p<0.01, n = 6/group). Additionally, high glucose levels did not impact the activities of the PDH-E1 subunit, citrate synthase (CS) or lactate dehydrogenase (LDH) in BeWo CT and SCT cells (**Table 3,** n = 6/group). Syncytialization additionally did not affect the enzyme activities of CS or LDH in BeWo trophoblasts (**Table 3**).

### HG-cultured BeWo trophoblast mitochondrial fission and fusion dynamics

**Fig 5:**
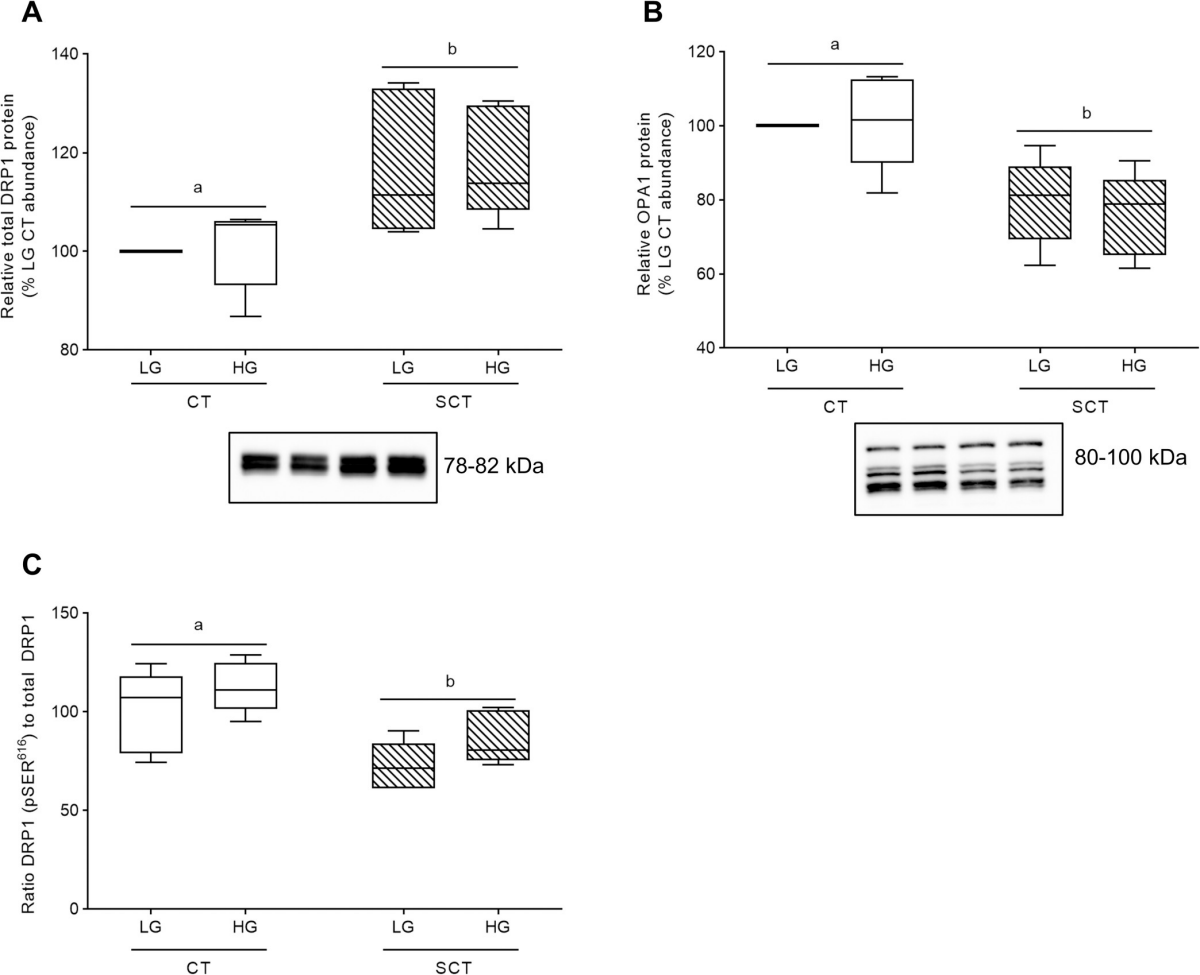
Syncytialization impacts mitochondrial dynamics in BeWo trophoblasts. Relative protein abundance of **(A)** total DRP1, **(B)** OPA1, and **(C)** ratio of pSER616 DRP1 to total DRP1. Different lower-case letters denote differentiation state-dependent differences in relative protein abundance between CT and SCT cultures (n = 5/group; Two-Way Randomized Block ANOVA (2WA), Sidak’s multiple comparisons test). Protein band density was normalized to total lane protein (ponceau) for statistical analysis and the total protein abundance data is presented as percent of LG CT abundance for visualization. Protein band density data from each membrane was normalized to the average LG CT protein band density prior to calculation of the phosphorylated protein abundance-to-total protein abundance ratio. Uncropped representative images of protein bands and ponceau staining of total lane protein are available in **S4 and S5 Figs in S1 File**.

Regardless of glucose level BeWo SCT cells displayed increased relative protein abundance of the mitochondrial fission marker DRP1 in conjunction with decreased relative protein levels of the mitochondrial fusion marker OPA1 compared to undifferentiated BeWo CT cells (**Fig 5A and 5B**; p<0.05, n = 5/group).

HG culture conditions additionally did not impact total protein abundance of OPA1 and DRP1 in BeWo trophoblasts (**Fig 5A and 5B**). There was a trend towards increased pSER^616^ phosphorylation of DRP1 in HG-cultured BeWo trophoblasts (mean 19% increase; **S3A Fig in S1 File**; p = 0.0632, n = 5/group) as well as reduced pSER^616^ DRP1 phosphorylation in syncytialized BeWo trophoblasts (**S3A Fig in S1 File**; p = 0.0924) when protein levels were normalized to total lane protein via ponceau stain. When the pSER^616^ DRP1 bands were expressed relative to total DRP1 protein levels, there was increased pSER^616^ DRP1:DRP1 levels in HG cultured trophoblasts (**Fig 5C**; p_glucose level_ <0.05, n = 5/group), and decreased pSER^616^ DRP1:DRP1 levels in syncytialized BeWo trophoblasts (**Fig 5C**; p_differentiation state_ <0.01). Post-hoc analysis identified trends towards significance in pSER^616^ DRP1-to-total DRP1 ratio levels in both BeWo CT cells (p = 0.0725) and SCT cells (p = 0.0512) (**Fig 5C**).

### HG culture conditions impact glycogen storage in BeWo trophoblasts

**Fig 6:**
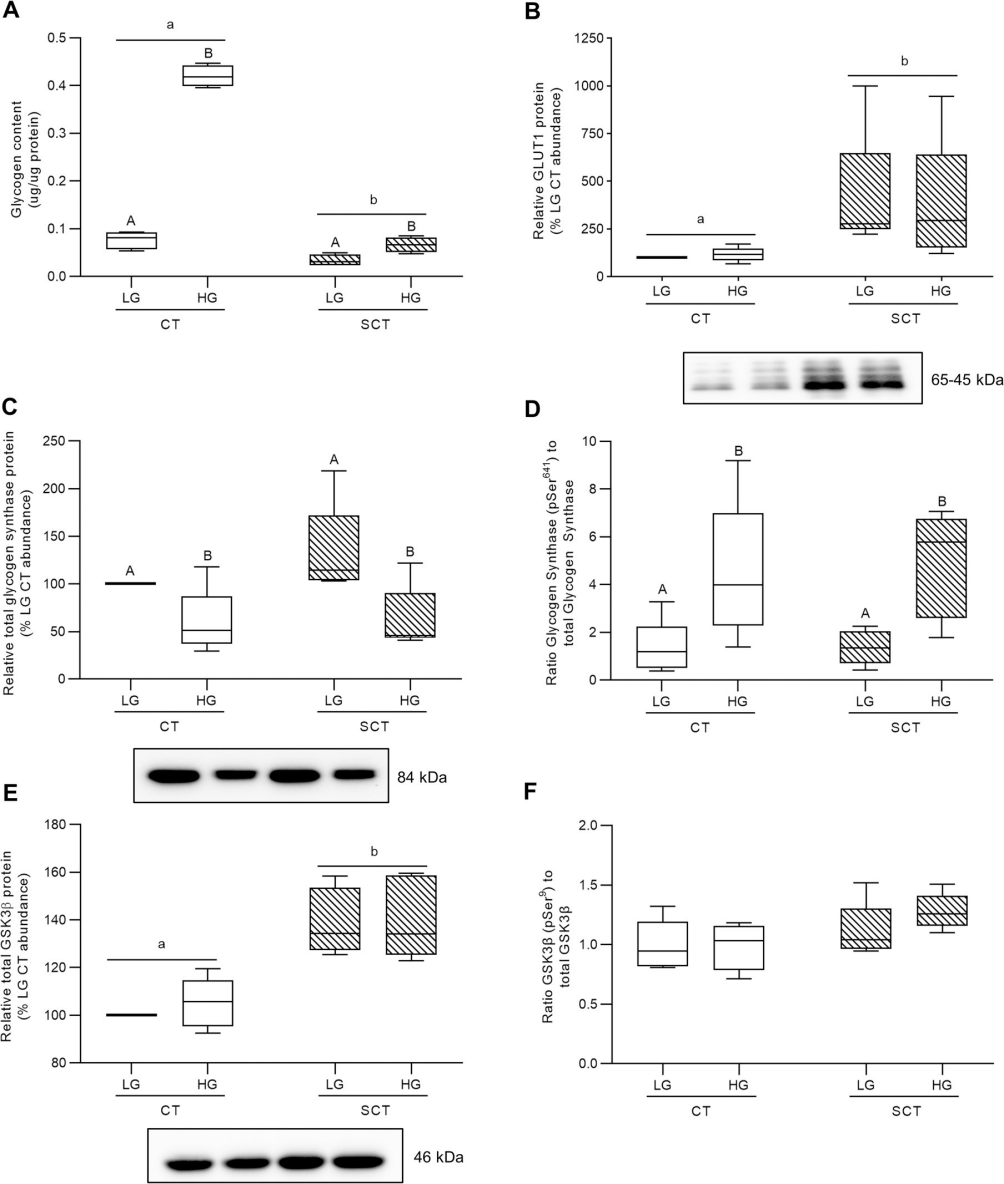
HG culture conditions impact glycogen storage and regulation in BeWo trophoblasts. **(A)** Glycogen content and relative protein abundance of **(B)** GLUT1 **(C)** glycogen synthase; **(D)** the pSer^641^ glycogen synthase-to-total glycogen synthase ratio; **(E)** GSK3β; and **(F)** the pSer^9^ GSK3β-to-total GSK3β ratio in HG-treated BeWo trophoblasts at T72H. Different lower-case letters denote differentiation state-dependent differences in protein abundance between CT and SCT cultures, and different upper-case letters denote glucose-level dependent differences within a differentiation state (Randomized-Block Two-Way ANOVA (2WA), and Sidak’s multiple comparisons test, p<0.05). Glycogen content data are presented as protein normalized glycogen abundance (μg glycogen per μg protein; n = 4/group). Protein band density was normalized to total lane protein (ponceau) for statistical analysis and the data is presented as percent of LG CT abundance for visualization (n = 5/group). Protein band density data from each membrane was normalized to the average LG CT protein band density prior to calculation of the phosphorylated protein abundance-to-total protein abundance ratio. Uncropped representative images of protein bands and ponceau staining of total lane protein are available in **S6 Fig in S1 File**.

HG culture conditions in both BeWo CT and SCT cells resulted in increased cellular glycogen content compared to respective differentiation state LG cultures (**Fig 6A**; p<0.0001; n = 4/group). Furthermore, the glycogen content in BeWo CT cultures was found to be greater than that of the SCT cultures (**Fig 6A**; p<0.0001). In addition, BeWo SCT cells displayed increased GLUT1 protein abundance compared to BeWo CT cells (**Fig 6B;** p<0.05), however, no glucose-dependent impacts to GLUT1 protein abundance were observed.

However, HG culture conditions were associated with reduced relative glycogen synthase protein abundance in both BeWo CT and SCT cells (**Fig 6C;** p<0.05, n = 5/group). HG cultured BeWo CT and SCT cells displayed increased total abundance of phosphorylated (pSer^641^) glycogen synthase (**S3B Fig in S1 File;** p<0.01, n = 5/group), and additionally differentiated BeWo SCT cells displayed increased phosphorylated (pSer^641^) glycogen synthase levels compared to undifferentiated CT cultures (**S3B Fig in S1 File;** p<0.01, n = 5/group). When phosphorylated Glycogen Synthase levels were expressed relative to total Glycogen Synthase levels, an increase in inhibitory phosphorylation was observed in both HG-cultured BeWo CT cells and SCT cells (**Fig 6D**; p<0.05, n = 5/group).

Furthermore, differentiated BeWo SCT cells were found to have increased protein levels of both GSK3β **(Fig 6E**; p<0.05, n = 5/group) and phosphorylated (pSer^9^) GSK3β (**S3C Fig in S1 File;** p<0.05, n = 5/group) compared to BeWo CT cultures. However, levels of phosphorylated (pSer^9^) GSK3β were found to not impacted by glucose culture condition when the protein levels were expressed as a phosphorylated protein abundance-to-total protein abundance ratio (**Fig 6F**; n = 5/group). HG culture conditions were not associated with alterations in the relative protein abundance of either GSK3β or phosphorylated (pSer^9^) GSK3β (**Fig 6E and 6F;** n = 5/group).

### HG culture conditions increase TG abundance in BeWo CT cells

**Fig 7:**
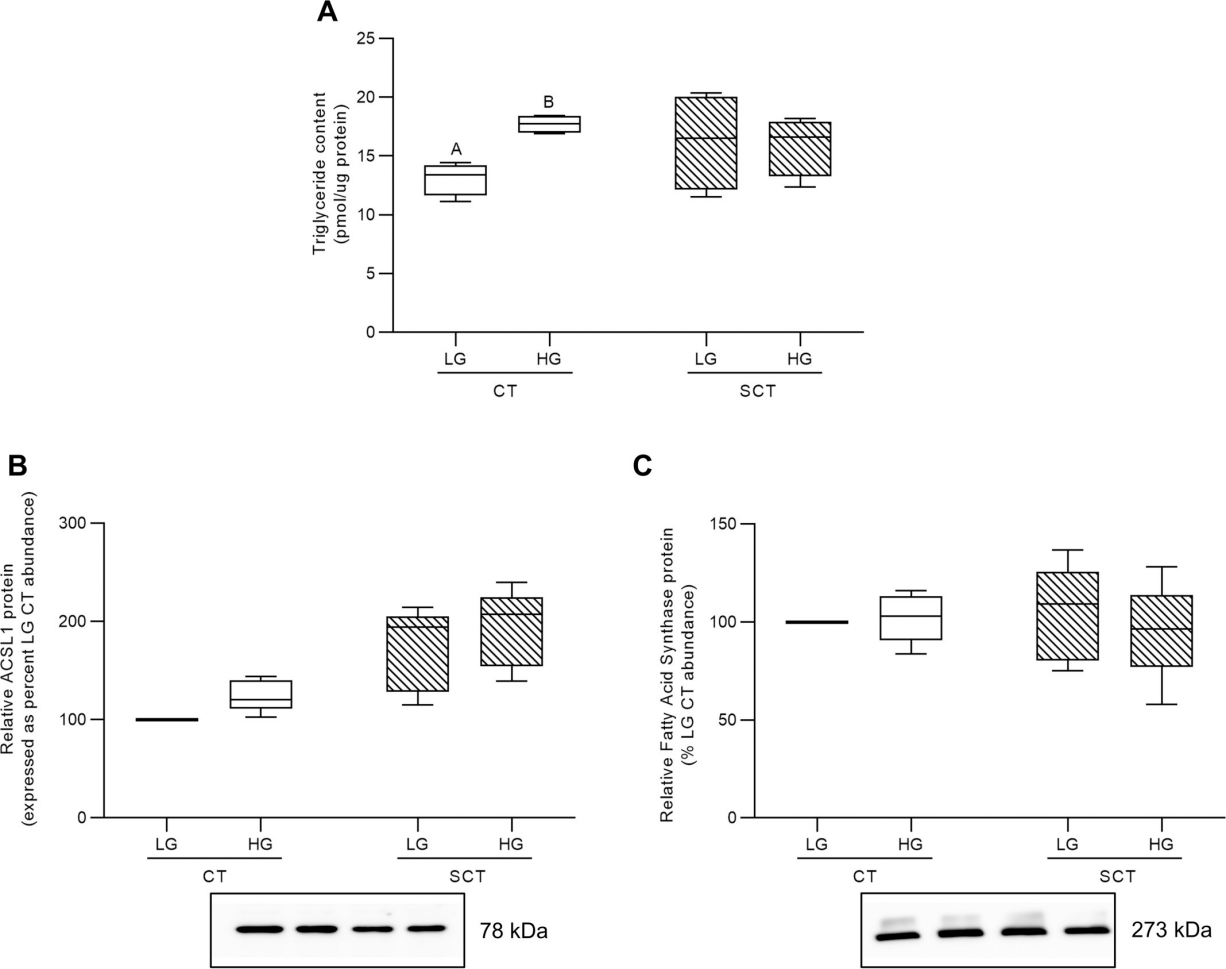
HG culture conditions impact triglyceride content in BeWo CT cells. **(A)** Triglyceride content and relative protein abundance of **(B)** ACSL1, and **(C)** FASN in HG-treated BeWo trophoblasts at T72H relative abundance. Different upper-case letters denote hyperglycemia-dependent differences between LG and HG treatments within each respective differentiation state (Two-Way Randomized Block ANOVA; Sidak’s multiple comparisons test, p<0.05). TG content data is presented as protein normalized TG abundance (pmol TG per μg protein; n = 4/group). Protein band density was normalized to total lane protein (ponceau) for statistical analysis and the data is presented as percent of LG CT abundance for visualization (n = 5/group). Uncropped representative images of protein bands and ponceau staining of total lane protein are available in **S4 Fig in S1 File**.

HG culture conditions were associated with increased triglyceride accumulation in BeWo CT cells, but not in BeWo SCT cells (**Fig 7A;** p<0.01, n = 4/group). Furthermore, BeWo syncytialization and HG culture conditions did not impact the relative abundance of ACSL1 protein, although there was a trend towards increased expression in both differentiated BeWo SCT cells and in HG-treated BeWo trophoblasts (**Fig 7B**; 2WA: glucose level p = 0.0894; 2WA: differentiation state p = 0.0695, n = 5/group). Finally, syncytialization and HG culture conditions did not impact the relative abundance of fatty acid synthase (FASN) in BeWo trophoblasts (**Fig 7C**).

### Transcriptomic profiling of HG-cultured BeWo CT cells

**Fig 8:**
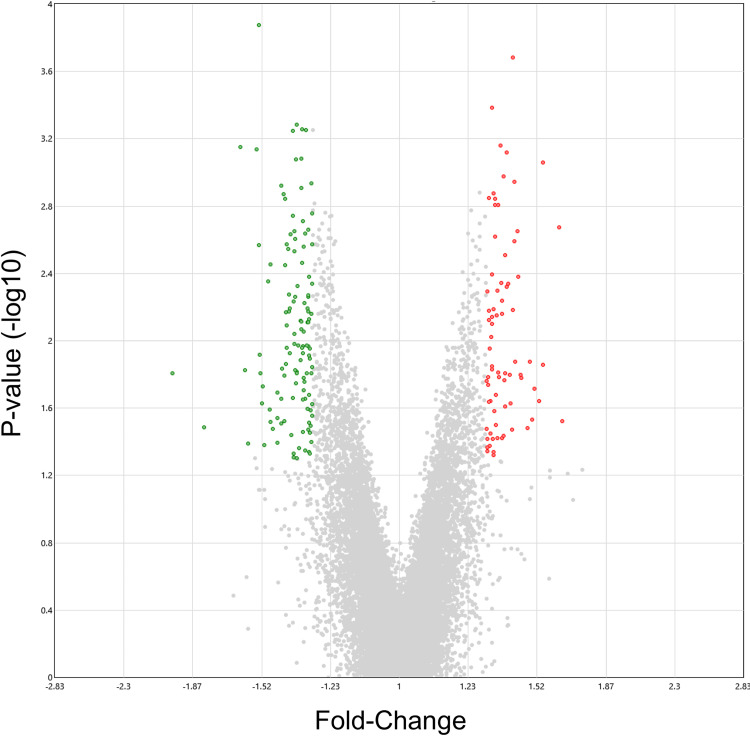
Volcano plot visualization of differentially expressed genes between LG and HG cultured BeWo CT cells. The volcano plot was generated to visualize differentially expressed genes in HG-cultured BeWo CT cells (≥ ±1.3-fold change, p<0.05, n = 5/group). Overall, 197 genes (122 up-regulated, and 75 down-regulated) were identified to be differentially expressed in HG-cultured BeWo CT cells. A summary list of the identified differentially expressed genes is available in **S3 Table in S2 File**. The x-axis indicates fold-changes vs LG BeWo CTs, and the y-axis indicates the p-value (-log10). The green dots represent statistically significant down-regulated genes, and the red dots represent statistically significant upregulated genes in HG-cultured BeWo CT cells.

HG-cultured BeWo CT cells displayed 197 differentially expressed genes (75 upregulated, 122 down-regulated) compared to LG BeWo CT cells (**S3 Table in S2 File,** ≥ ±1.3-FC vs LG CT). The volcano plot (**Fig 8**) and 2D hierarchical clustering heatmap (**S7 Fig in S1 File**) were constructed to visualize the degree of gene expression differences between LG and HG cultured BeWo CT cells.

**Fig 9:**
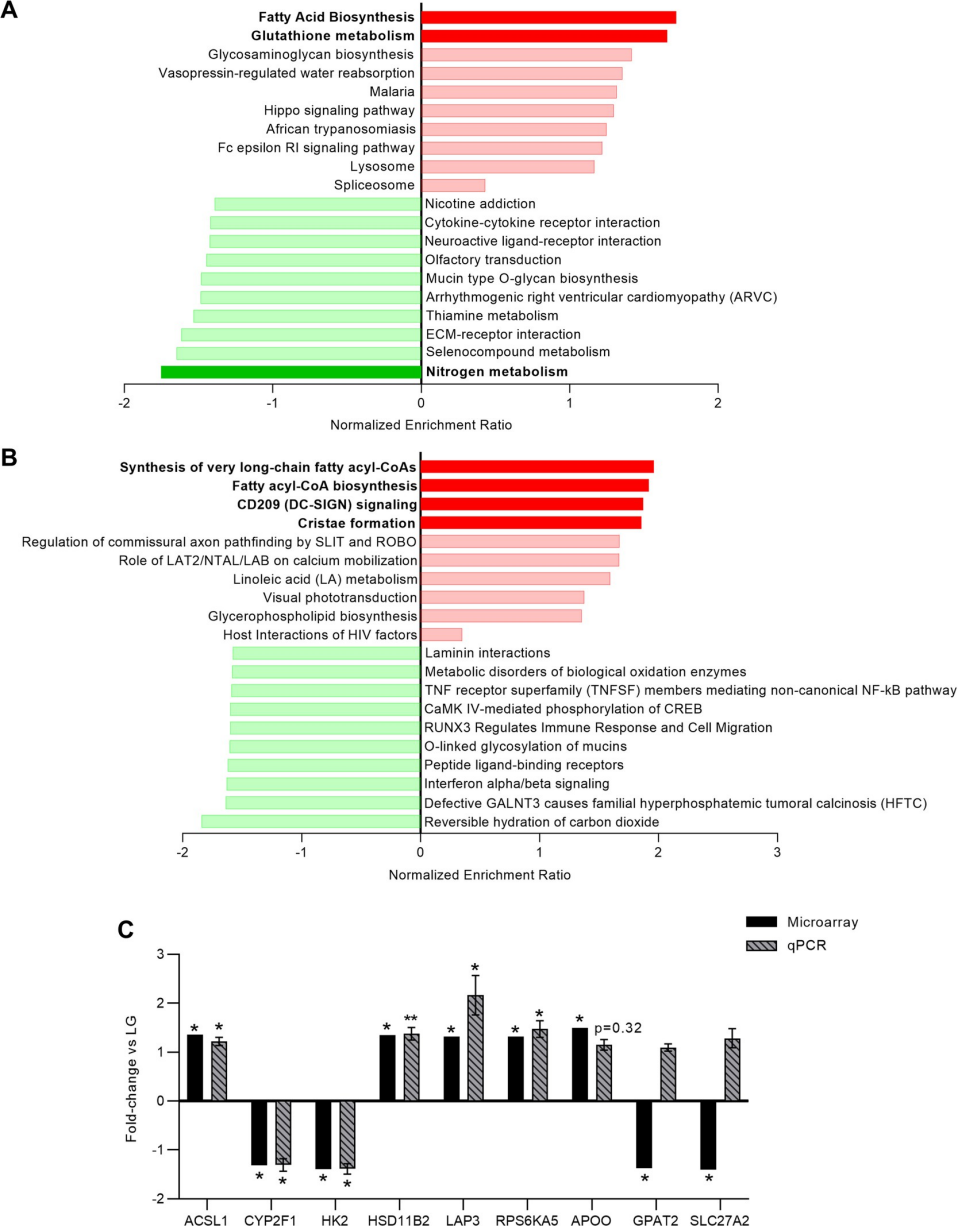
Gene set enrichment analysis of differentially expressed genes and RT-qPCR validation of microarray gene changes. **(A)** Top 10 up-regulated and down-regulated KEGG gene sets and **(B)** top 10 up-regulated and down-regulated Reactome gene sets (by normalized enrichment ratio) in HG-cultured BeWo CT cells. Green bars represent down-regulated gene sets and red-bars represent up-regulated gene sets, with dark green bars representing significantly down-regulated and dark red bars representing significantly up-regulated gene sets (FDR-p<0.25). The transcripts identified in the microarray were ranked by signal-to-noise ratio using the Gene Set Enrichment Analysis Software, and enrichment analysis was performed using WebGestalt. **(C)** RT-qPCR was utilized to validate the differential expression of ACSL1, CYP2F1, HK2, HSD11B2, LAP3, and RPS6KA5 in HG-cultured BeWo CT cells. Data was analyzed via paired t-test (*p<0.05, **p<0.01, n = 9-10/group), and data expressed as fold-change vs LG BeWo CT (mean ± SEM). Down-regulated genes ratios were inversed and expressed as a negative (e.g 0.5-fold is expressed as a minus-2 fold-change).

Gene-Set Enrichment Analysis (GSEA) was then performed using all transcripts identified in the microarray panel, with genes ranked according to their signal-to-noise ratio. A list of all genes and their associated signal-to-noise rank score is available in **S4 Table in S2 File**. Enrichment analysis was performed using the KEGG, Reactome, and Wikipathways functional gene sets, as well as with the Gene Ontology (GO) biological processes and molecular function gene sets. Significant enrichment in KEGG pathways was found in the HG-cultured BeWo CT cells that were associated with an upregulation in the “Fatty Acid Biosynthesis” gene set (the genes involved were: ACSL1 (+1.36 FC), ACACB, ACSL5, and ACSBG1), and the “Glutathione Metabolism” gene set (the gene involved were: LAP3 (+1.32 FC), GPX5, CHAC1, GGT7, MGST1, ANPEP, GSTA3, GLCM, GSTO1, and NAT8) (FDR-p<0.25, **Fig 9A**). A significant down-regulation in the KEGG “Nitrogen Metabolism” gene set (the genes involved were: CA5A (-1.53 FC), CA1, CA6, CA7, and CA13) was also observed (FDR-p<0.25, **Fig 9A**).

Analysis using the Reactome functional gene sets was associated with significant up-regulation in the “Synthesis of very long-chain fatty acyl-CoAs” gene set (the genes involved were: ELOVL7, ACSL1 (+1.36 FC), ELOVL4, ELOVL3, ACSL5, and ACSBG1); the “Fatty acyl-CoA biosynthesis” gene set (the genes involved were: ELOVL7, ACSL1 (+1.36 FC), ELOVL4, ELOVL3, ACSL5, CBR4, and ACSBG1); the “CD209 (DC-SIGN) signaling” gene set (the genes involved were: CD209, RPS6KA5 (+1.32 FC), FYN, ICAM3, PRKACB, HRAS, and ICAM2); and the “Cristae formation” gene set (the genes involved were: APOO (+1.5 FC), and CHCHD6) (FDR-p<0.25, **Fig 9B**). No significant enrichment was found in the Wikipathways functional gene sets (**S8A Fig in S1 File)**.

GSEA analysis in the Gene Ontology (GO) databases revealed a significant down-regulation in the GO “Oxygen Binding” molecular pathway database (the genes involved were: CYP2A16, CYP1A1, CYP2F1 (-1.32 FC), ADGB, ALB, CYP2A7, NGB, NOX4, CYP17A, and HBM) (FDR-p<0.25; **S8B Fig in S1 File**). There was no significant enrichment in the GO biological processes database (**S8C Fig in S1 File)**.

The expression of DEGs identified to be involved in significantly upregulated and down-regulated functional pathways (ACSL1, APOO, CA5A, CYP2F1, DGKG, GPAT2, LAP3, LIPF, RPS6KA5) were subsequently validated via RT-qPCR. Individual DEGs involved in cellular metabolic processes (HSD11B2, HK2, and SLC27A2) were also identified from the microarray panel and selected validation via RT-qPCR. The transcripts for the DEGs: CA5A, DGKG, LIPF could not be detected via RT-qPCR in BeWo lysates, and thus the differential expression of these targets could not be validated.

However, the differential expression of ACSL1 (+1.36 FC microarray; +1.22 FC RT-qPCR); CYP2F1 (-1.32 FC microarray; -1.31 FC RT-qPCR); HSD11B2 (+1.35 FC microarray; +1.38 FC RT-qPCR); HK2 (-1.39 FC microarray; -1.39 FC RT-qPCR); LAP3 (+1.32 FC microarray; +2.17 FC RT-qPCR); and RPS6KA5 (+1.32 FC microarray; +1.47 FC RT-qPCR) in HG-cultured BeWo CT cells were validated via RT-qPCR. (**Fig 9C**) The differential expression of: APOO (+1.50 FC microarray; +1.15 FC, p>0.05 RT-qPCR); GPAT2 (-1.37 FC microarray; +1.10 FC, p>0.05 RT-qPCR; and SLC27A2 (-1.40 FC microarray; +1.29 FC, p>0.05 RT-qPCR) in HG-cultured BeWo CT cells could not be validated via RT-qPCR (**Fig 9C**).

### Impacts of HG culture conditions on the metabolome of BeWo CT cells

On average 6541 ± 42 (mean ± SD) metabolite peak pairs were measured in each sample. A summary of the metabolites identified in all tiers is available in **S5 Table in S2 File.** Of these peak pairs, 179 were positively identified in tier 1 and 602 peak pairs were identified with high confidence in tier 2. Of these identified peak pairs, 4 from tier 1, 89 from tier 2, and 627 from tier 3 were found to be differentially abundant (≥ ±1.5 FC, raw-p<0.05) between HG and LG cultured BeWo CT cells. A list of all differentially abundant metabolites between LG and HG-cultured BeWo CT cells is available in **S6 Table in S2 File**.

**Fig 10:**
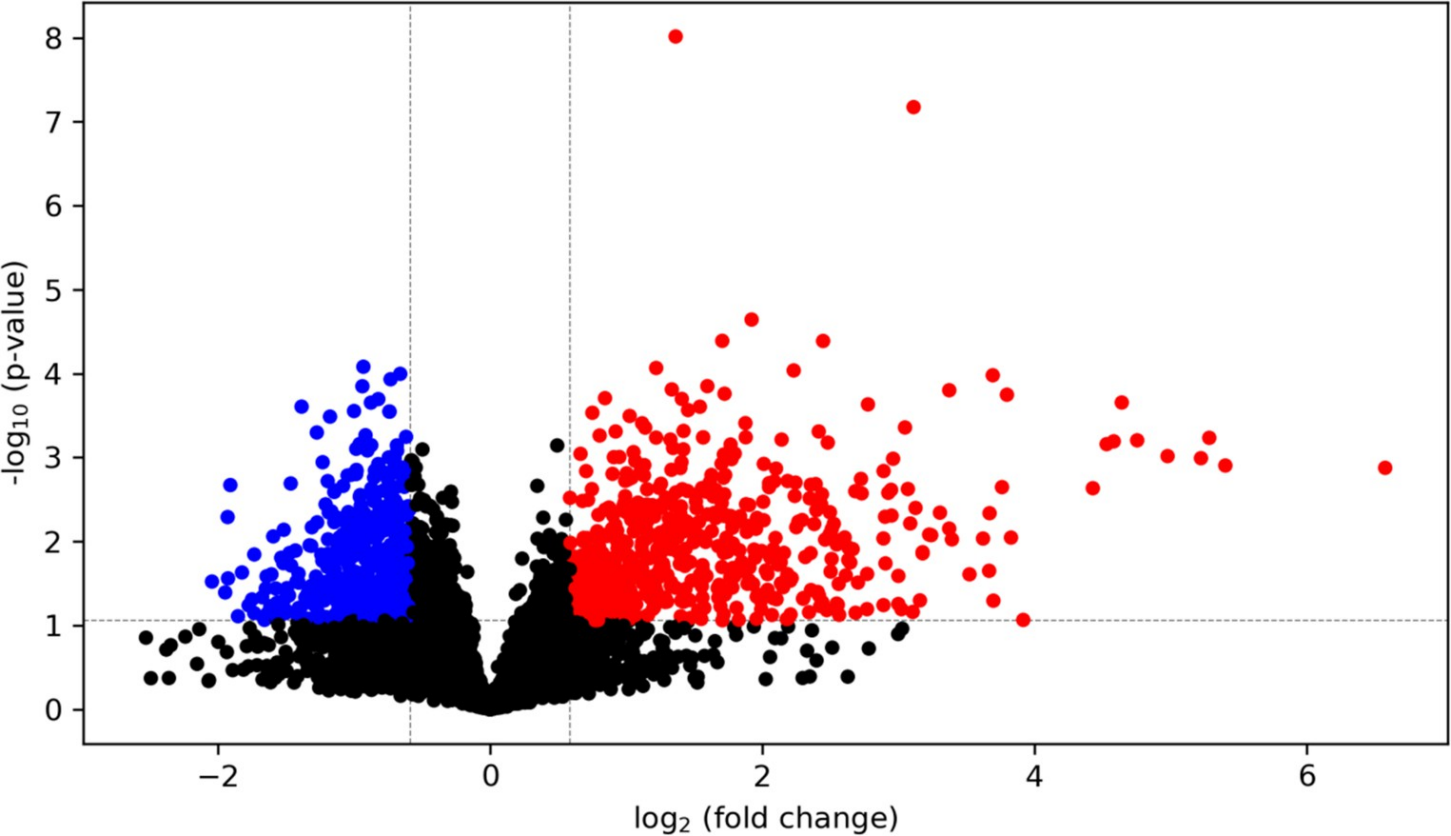
Visualization of differentially abundant metabolites in HG-cultured BeWo CT cells. **A** volcano plot was constructed to visualize the differentially abundant metabolites in HG-cultured BeWo CT cells ((≥ ±1.5-fold change, p<0.05, n = 5/group). Overall, 720 metabolites were found to be differentially abundant in HG-cultured BeWo CT cells. Of these metabolites, 4 were identified in tier 1 (all increased in abundance) and 89 were identified in tier 2 (22 decreased in abundance, and 67 increased in abundance) with high confidence. The x-axis indicates log_2_(fold-change) vs LG BeWo CTs, and the y-axis indicates the p-value (-log10). The red dots represent significantly increased metabolites, and the blue dots represent significantly decreased metabolites in HG-cultured BeWo CT cells.

Differentially abundant metabolites were subsequently visualized via volcano plot (±1.5 FC, p<0.05, **Fig 10**) and heat map (**S9 Fig in S1 File**). Additionally, the degree of differences in metabolite profiles between HG and LG-cultured BeWo CT cells was visualized by unsupervised principal component analysis (PCA) 2D plot as well as supervised partial least squares discriminant analysis (PLS-DA) scores plot (**S10A and S10B Fig in S1 File**).

**Fig 11:**
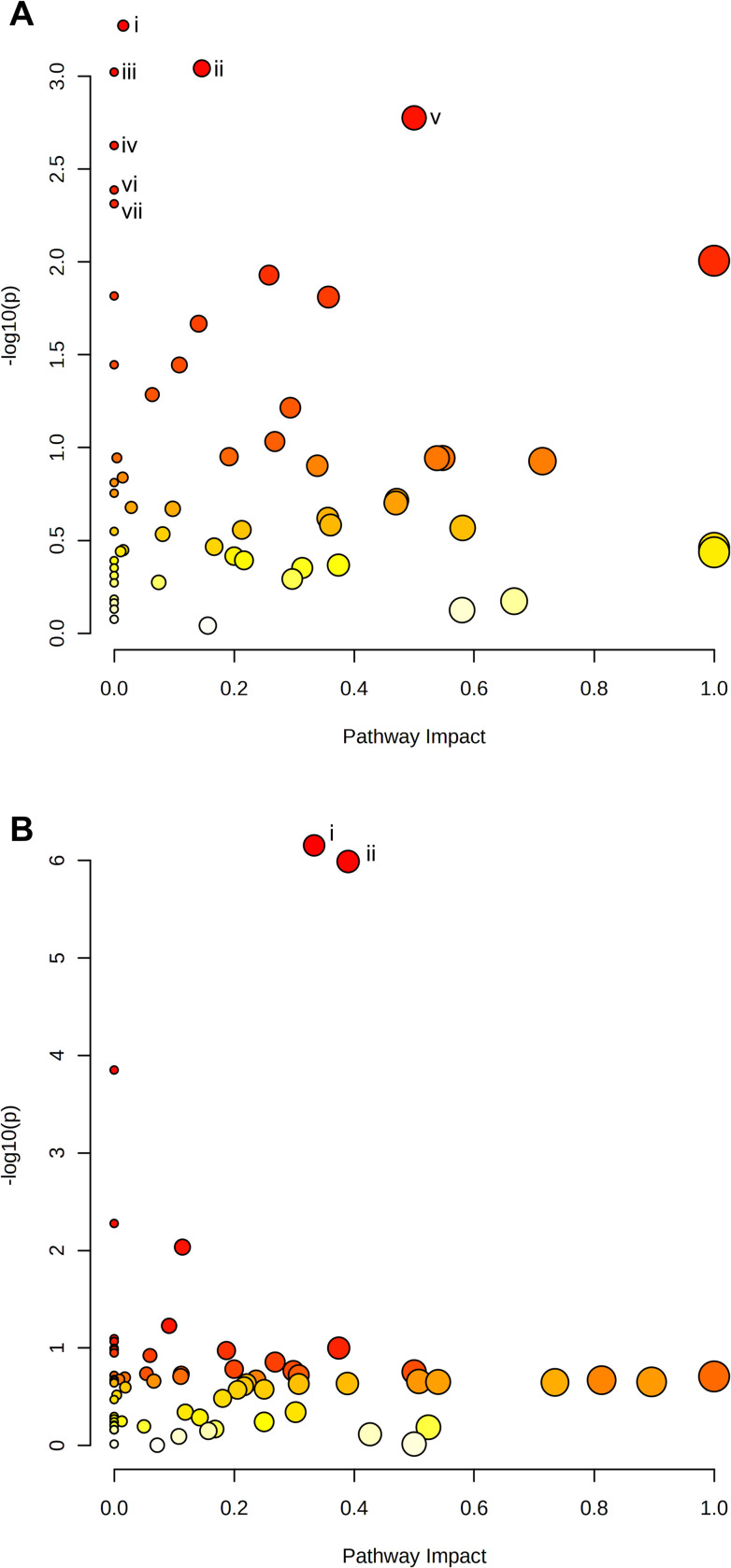
Pathway analysis of metabolites identified in tiers 1 and 2. Identified peak pairs from tiers 1 and 2 were imported into MetaboAnalyst v5.0 for analysis of enriched KEGG and Small Molecule Pathway Database (SMPDB) pathways. A scatterplot was created to visualize identified pathways with pathway impact (ratio of identified metabolites to total metabolites in the pathway) on the x-axis and p-value (-log10) on the y-axis. Each individual node represents a unique KEGG pathway, with the node size corresponding to pathway impact and node colour corresponding to significance level. Pathways with a false discovery rate p<0.05 were determined to be significant and were labelled with roman numerals. **(A)** The KEGG database pathways involving: (i) Glycolysis/Gluconeogenesis, (ii) Pyruvate Metabolism, (iii) Drug Metabolism–Cytochrome P450, (iv) Ascorbate and Aldarate Metabolism, (v) Riboflavin Metabolism, (vi) Fatty Acid Biosynthesis, and (vii) Synthesis and Degradation of Ketone Bodies pathways were significantly enriched in HG-cultured BeWo CT cells. **(B)** The SMPDB pathways involving: (i) the Glycerol Phosphate Shuttle; (ii) Glycerolipid Metabolism; and (ii) Riboflavin Metabolism were identified to be significantly enriched in the HG-cultured BeWo CT cells.

The KEGG database pathways involving: glycolysis/gluconeogenesis (associated with significantly increased lactate (+2.72 FC) levels, and significantly decreased acetaldehyde (+2.72 FC) levels); pyruvate metabolism (associated with increased lactate (+2.72 FC) levels, and decreased acetaldehyde (-1.20 FC) levels); drug metabolism–cytochrome P450 (associated with increased 2-hydroxyiminostilbene (+2.52 FC) and 3-carbamoyl-2-phenylpropionic acid (+5.42 FC) levels); ascorbate and aldarate metabolism (associated with increased L-gulonolactone (+2.46 FC) levels); riboflavin metabolism (associated with increased riboflavin (+3.68 FC) levels); fatty acid biosynthesis (associated with increased malonate (+3.74 FC) levels); as well as synthesis and degradation of ketone bodies (associated with increased (R)-3-hydroxybutanoate (+1.60 FC) levels) were identified to be significantly enriched in HG-cultured BeWo CT cells (FDR-corrected p<0.05, **Fig 11A**).

The SMPDB pathways involving: the Glycerol Phosphate Shuttle associated with significantly increased p-Benzoquinone (+3.78 FC) levels); Glycerolipid Metabolism (associated with significantly increased p-Benzoquinone (+3.78 FC) levels); and Riboflavin Metabolism (associated with significantly increased riboflavin (+3.68 FC) levels) were identified to be significantly enriched in the HG-cultured BeWo CT cells (FDR-corrected p<0.05; **Fig 11B**).

### Integration of HG-cultured BeWo cytotrophoblast transcriptome and metabolome signatures

**Fig 12:**
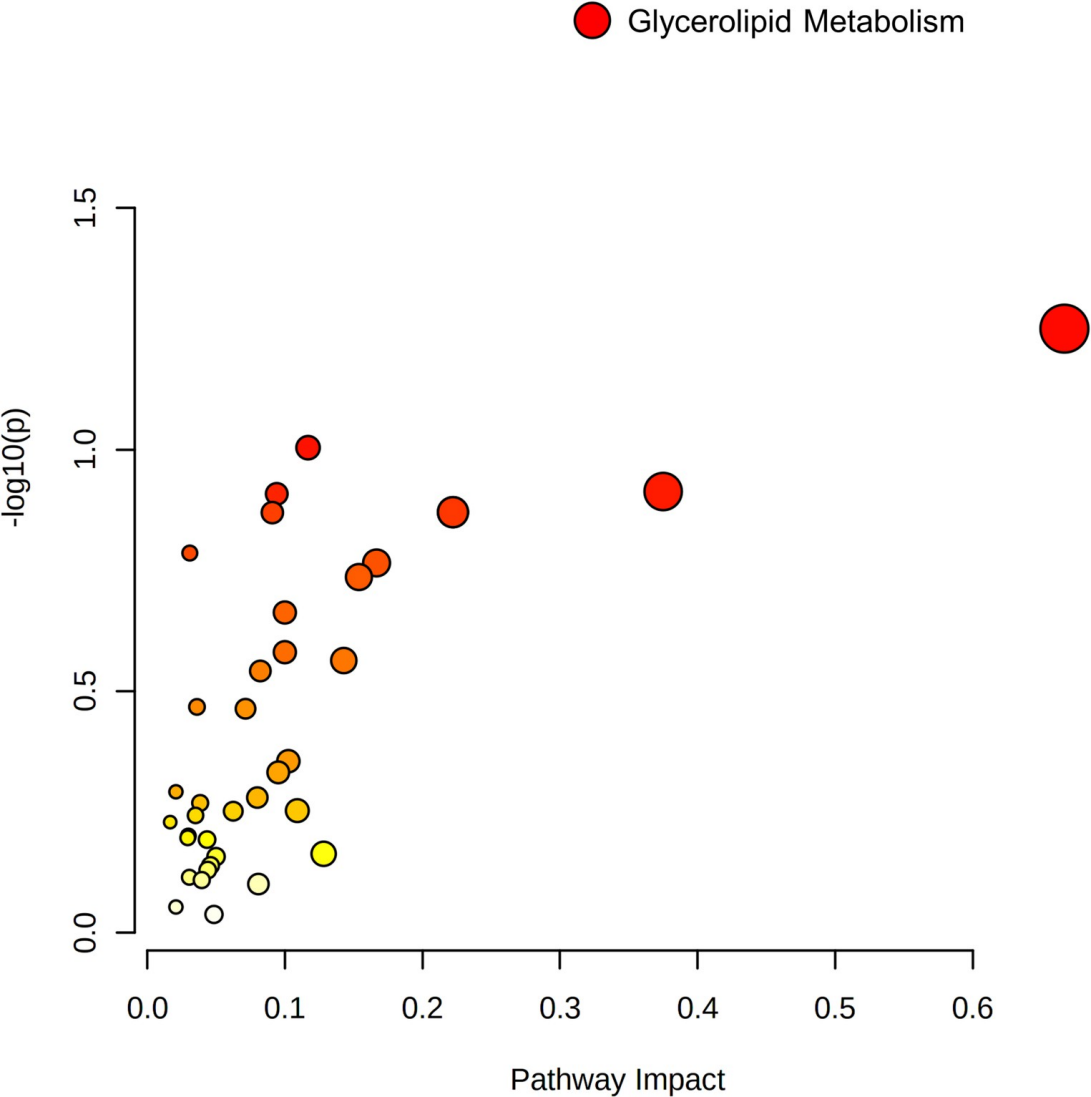
Joint pathway analysis integration of HG-cultured BeWo CT cell transcriptome and metabolome profiles. Differentially expressed gene and differentially abundant metabolite lists were imported into the MetaboAnalyst v5.0 Joint Pathway Analysis tool for transcriptome-metabolome integration analysis. The differentially expressed gene and differentially abundant metabolite lists were pooled into a single query for KEGG pathway over-representation analysis. A scatterplot was created to visualize identified pathways with pathway impact (ratio of identified metabolites to total metabolites in the pathway) on the x-axis and p-value (-log10) on the y-axis. Each individual node represents a unique KEGG pathway, with the node size corresponding to pathway impact and node colour corresponding to significance level. The (i) Glycerolipid metabolism pathway was found to be enriched in the HG-cultured BeWo CT cells, although this enrichment was only associated with differential gene expression, and not with simultaneous altered metabolite levels (raw-p <0.05).

Differentially abundant metabolites and DEGs were pooled into queries for KEGG pathway enrichment analysis. The KEGG Glycerolipid metabolism pathway was enriched in the HG-cultured BeWo CT cell datasets, however only differentially expressed genes (LIPF (+1.30 FC); DGKG (+1.31 FC); and GPAT2 (-1.37 FC)), and not differentially abundant metabolites were found to be involved in this pathway (**Fig 12;** raw-p<0.05).

## Discussion

This study aimed to expand upon current published literature [22,30,31,34] and more thoroughly explore the independent impacts of hyperglycemia on placental villous trophoblasts by characterizing nutrient storage and mitochondrial respiratory activity in BeWo trophoblasts following a relatively prolonged 72-hour HG-exposure (25 mM glucose). While previous studies utilizing BeWo trophoblasts have primarily highlighted the impacts of high glucose exposure on undifferentiated CT cells [30,31,34] the current study is strengthened through the combined examination of both undifferentiated CT cells and differentiated SCT cells. The more chronic 72-hour culture protocol as utilized in this study also allowed for exposure of villous trophoblast cell populations to hyperglycemia prior to and during differentiation, analogous to *in vivo* villous trophoblast layer development whereby progenitor CT cells are pre-exposed to dietary nutrients prior to and during syncytialization. More importantly, the specific use of functional readouts of metabolic and mitochondrial activity (including the Seahorse XF Mito and Glycolysis Stress Tests) in this study provided a more in-depth insight into the real-time metabolic function of live CT and SCT cells following a prolonged glucose challenge. Subsequently, the current study utilized a multi-omics research approach and described transcriptomic and metabolomic signatures of HG-exposed BeWo CT cells, allowing a thorough characterization of the glucose-mediated alterations to placental metabolism. Overall, the data presented in this study demonstrated that a 72-hour exposure to hyperglycemia, increased glycogen and triglyceride stores as well as modulated the transcriptomic and metabolomic signatures as well of BeWo trophoblast cells. However, impairments in functional readouts of BeWo trophoblast mitochondrial respiration were not observed following 72-hours of HG-exposure.

### Hyperglycemia and nutrient stores in BeWo trophoblasts

As previously highlighted, DM during pregnancy is associated with aberrant nutrient storage in the villous trophoblast layer of the placenta [18–23]. Our results demonstrated that HG-culture conditions directly promoted increased glycogen content in both BeWo CT and SCT cells, and additionally, that BeWo CT cells have a greater glycogen storage potential than SCT cells. It is important to note that these differentiation state-dependent trends in glycogen content are consistent with previous reports from primary human placental tissue which described that glycogen storage predominately occurs within the CT cells of the placenta [24,50,51]. Increased accumulation of glycogen stores in diabetic placentae has been thought to be a mechanism by which the placenta limits materno-fetal glucose transfer in times of nutrient overabundance to limit fetal overgrowth [24,25]. However, high-glucose exposure in both BeWo CT and SCT cells was also associated with reduced relative protein abundance of glycogen synthase as well as with increased inhibitory Ser^641^ phosphorylation [52] of glycogen synthase. As there were no glucose-mediated differences in the protein abundance of GSK3β or the inhibitory Ser^9^ phosphorylation [53] of GSK3β, the increased inhibitory phosphorylation of glycogen synthase in HG-cultured BeWo trophoblasts likely occurs via a GSK3β-independent mechanism.

Overall, these data highlighted that following 72-hours of hyperglycemia BeWo trophoblast cells, despite having elevated stores of glycogen, have a diminished capacity to continue to store excess glucose as glycogen. The changes could reflect the presence of a negative feedback mechanism that acts to prevent excessive glycogen accumulation in placental villous trophoblasts, similar to the feedback mechanisms described in skeletal muscle cells [54]. Excessive glycogen levels have previously been associated with liver inflammation in rodents [55] and reduced lifespan in *Caenorhabditis elegans* [56], which suggests that glycogen accumulation is damaging. Therefore, the diminished glycogen storage capacity in placental trophoblasts in response to prolonged high glucose could act in a protective manner. However, these changes in glycogen storage dynamics in placental trophoblasts following sustained HG-exposure could also lead to increased materno-fetal glucose transport. This may be an important alteration to placental glucose processing that helps facilitate the development of fetal macrosomia in diabetic pregnancies [57].

In addition to increased glycogen stores, our study also demonstrated that BeWo CT cells, but not SCT cells, have increased triglyceride content in response to excess glucose exposure. These data were consistent with previous reports that have highlighted an increased accumulation of triglyceride species in placental explants from healthy pregnancies under high glucose conditions [22]. These glucose-mediated changes in trophoblast triglyceride abundance will likely modulate materno-fetal nutrient transfer and could have downstream impacts on fetal growth and development. The current study further identified that there were no differentiation state-dependent differences in triglyceride abundance in BeWo trophoblast cells, similar to what has been reported in freshly isolated villous trophoblast samples [58]. Previous readouts from primary placenta samples have highlighted that lipid esterification processes occur primarily within villous CT cells, and it has been suggested that any lipid droplets present in primary SCT cells are remnants from esterification process that occurred prior to syncytialization [58]. We speculate that a similar reduction in esterification activity also occurs in BeWo SCT cells and may explain the absence of a HG-mediated difference in triglyceride content in our BeWo SCT cells. Future studies utilizing functional readouts of lipid esterification activity may be needed to better elucidate the mechanisms underlying differences in TG abundance between undifferentiated BeWo CT cells and differentiated BeWo SCT cells.

### Hyperglycemia and metabolic function in BeWo trophoblasts

The current study demonstrated that cellular mitochondrial respiratory activity (measured by the Seahorse XF Mito Stress Test) as well as that the activities of ETC complexes I and II are not impacted in BeWo trophoblasts cultured under hyperglycemia at 72H. Additional analysis of metabolic function via the Seahorse Glycolysis Stress Test and activity assays for LDH and CS enzymes further indicated that isolated hyperglycemia does not impact functional aspects of metabolism in BeWo trophoblasts. Overall, our data suggests that hyperglycemia alone may not directly facilitate the impairments in mitochondrial respiratory activity that have previously been observed in DM-exposed primary placental samples [27–29].

Previous studies in other cell preparations, however, have demonstrated that the impacts of HG-culture conditions on mitochondrial respiratory function are dependent on the length of high-glucose exposure. For example, human kidney tubule (HK-2) cells have displayed reduced basal and maximal mitochondrial respiratory activity only when cultured under HG-conditions (25 mM) for at least 4 days [59]. Further, mitochondrial respiratory activity in kidney glomerular (HMC) cells was not impacted prior to 8 days of high glucose exposure [59]. Likewise, mitochondrial activity of human umbilical cord endothelial (EA.hy926) cells was not impaired after 3 days of high glucose (25 mM) treatment, but was reduced after 6 days of HG-culture conditions, and this impairment in mitochondrial function was sustained through 9 days of high glucose exposure [60]. Overall, these studies suggest that there are time-course dependent factors that influence whether hyperglycemia impacts cellular mitochondrial respiratory activity. Thus, the conclusions of the current study may be limited due to the single timepoint utilized for all analyses of metabolic function. It is possible that prolonging HG-culture conditions in BeWo trophoblasts could ultimately lead to impaired mitochondrial function, as was observed in HK-2, EA.hy926, and HMC cells. The impacts of a longer duration of high glucose treatment, along with sequential sampling over a time course, in BeWo cells may need to be explored in future investigations.

In addition, previous research has highlighted that hyperglycemia negatively regulates mitochondrial function in some cell types by altering mitochondrial fusion (regulated by OPA1) and fission (regulated by DRP1) dynamics leading increased mitochondrial fractionation [61–64]. In the current study, we observed a significant increase in the pSER^616^ DRP1-to-total DRP1 ratio in HG-cultured BeWo trophoblasts. This post-translational modification of DRP1 is associated with increased mitochondrial translocation of DRP1 that promotes increased mitochondrial fission [65]. These underlying changes in mitochondrial physiology are important, as increased mitochondrial fission has been associated with increased cellular oxidative stress, and impaired insulin sensitivity, and ultimately mitochondrial respiratory dysfunction [66,67]. Further, these data may indicate that underlying aspects of trophoblast mitochondrial function are negatively regulated by hyperglycemia, despite concurrent our observation that global, real-time measures of BeWo mitochondrial respiratory activity were not impacted. Further, we speculate this could indicate that HG-exposed BeWo trophoblasts are transitioning towards mitochondrial dysfunction mediated by increased mitochondrial fission, comparable to the outcomes reported in skeletal muscle following high fat exposure [66].

While functional global readouts of BeWo trophoblast mitochondrial function were not impacted in response to hyperglycemia, we did observe impaired mitochondrial respiratory activity in differentiated BeWo SCT cells. Syncytialization of BeWo trophoblasts was associated with reduced mitochondrial spare respiratory capacity, reduced coupling efficiency, concomitant with reduced activity of ETC complex II and reduced protein expression of ETC complex IV. Furthermore, BeWo SCT cells displayed increased DRP1 protein abundance, an increased pSER^616^ DRP1-to-total DRP1 ratio, and decreased OPA1 protein abundance suggestive of increased mitochondrial fractionation. These data may indicate that alterations in mitochondrial dynamics underlies the observed functional differences in mitochondrial respiration between BeWo CT and SCT cells. Previous work by our research group has likewise highlighted that BeWo CT cells are overall more metabolically active than BeWo SCT cells [37], and similar trends have been reported in cultured PHT cells [28,68]. As undifferentiated BeWo CT cells display greater metabolic activity and greater alterations to nutrient stores in response to hyperglycemia than in differentiated SCT cells, we speculated that alterations in transcriptome and metabolome profiles in response to high glucose culture conditions would be more prevalent in these progenitor cells. Thus, the current study sought to examine global gene expression as well as global metabolite abundance solely in HG-cultured BeWo CT cells to further elucidate mechanisms underlying altered placental metabolic function in response to hyperglycemia.

### Transcriptomic analysis of HG-cultured BeWo CT cells

Overall, we identified 197 differentially expressed genes (122 up-regulated, and 75 down-regulated; ≥ ±1.3 FC) in BeWo CT cells cultured under hyperglycemia for 72H. However, previous reports in BeWo CT cells demonstrated more substantial variations in gene expression (>5000 DEGs) between BeWo trophoblasts cultured under similar high and low glucose conditions [30]. The differences between the current study and previous reports may be due in part to differences in study design (pooling samples for arrays vs independent arrays for each sample), cell media formulation (DMEM-F12 vs F12K) as well as length of hyperglycemic exposure (48H vs 72H). Despite these differences in the number of differentially expressed genes, our study did align with this previous transcriptomic report, and further demonstrated that elevated glucose levels impact the expression of genes involved in functional pathways involving metabolic processes [30].

Specifically in the current study, using a Gene Set Enrichment Analysis (GSEA) approach, we highlighted an enrichment in metabolic functional and molecular pathways relating to glutathione metabolism (associated with increased LAP3 expression), fatty acid synthesis (associated with increased ACSL1 expression), CD209 signaling (associated with increased RPS6KA5 expression), and oxygen binding (associated with reduced CYP2F1 expression) in HG-cultured BeWo CT cells. Additionally, we validated the differential expression of genes involved in glucose metabolism (reduced HK2 expression) and glucocorticoid metabolism (increased HSD11B2 expression) in our high-glucose exposed cultures.

Of particular interest was the increased mRNA expression of *ASCL1* in BeWo CT cells, a gene involved in functional gene sets associated with fatty acid and lipid synthesis. Previous studies have highlighted that *ACSL1* is involved in lipid synthesis in various tissues and that knockdown of *ACSL1* is associated with reduced triglyceride and lipid droplet abundance [69–72]. More importantly, cells transfected to overexpress *ACSL1* have been found to have increased triglyceride accumulation [72–74]. We speculate that *ACSL1* is also important in the trafficking of lipid species to lipid droplets in trophoblast cells and the increased expression of this transcript may underlie the glucose-induced accumulation of triglyceride species that was observed in our BeWo CT cultures. Thus, *ACSL1* may be a regulator of trans-placental lipid transport, and its altered expression in response to elevated glucose could impact fetal growth by reducing materno-fetal lipid transport. In contrast, the observed reduction in Hexokinase-2 (HK2, the first enzyme in the placental glycolysis metabolic pathway [27]) transcript abundance could indicate that glycolysis is down-regulated in trophoblasts exposed to hyperglycemia. The reduced expression of glycolytic enzymes combined with a reduced capacity for glycogen storage (reduced glycogen synthase expression and increased inhibitory phosphorylation of glycogen synthase) could facilitate increased materno-fetal glucose transport. The overall impacts of hyperglycemic exposure on transplacental nutrient transport will need to be examined in more depth in future experiments (perhaps through the use of trans well culture systems) to elucidate how fetal nutrient delivery is modulated in response to these changes in placental nutrient handling.

The results from the current study additionally demonstrated that hyperglycemia is an important regulator of the expression of placental *HSD11B2*, an enzyme involved in the metabolism and inactivation of the glucocorticoid cortisol. In normal pregnancies, cortisol is thought to be involved in mediating the physiological increase in maternal insulin resistance that is necessary to support fetal growth [75,76], however, circulating cortisol levels may be pathologically elevated in some GDM pregnancies [77]. Interestingly, placentae from GDM pregnancies have been found to have increased expression of *HSD11B2* leading to increased cortisol inactivation [78]. As, elevated cortisol levels in fetal circulation have been associated with impaired brain development processes [79], increased placental *HSD11B2* expression in GDM pregnancies may act in a protective manner to limit fetal glucocorticoid exposures. However, as cortisol also activates the glucocorticoid receptor leading to modulation of gene transcription [80,81], alterations in placental cortisol metabolism may have downstream consequences on placental function through altering gene expression. Overall, it remains poorly understood whether these glucose-mediated impacts to placental cortisol metabolism act in a protective or detrimental manner.

These transcriptomic readouts may also highlight underlying alterations to stress responses in BeWo trophoblast cells exposed to hyperglycemia. For example, we observed a significant up-regulation in the CD209 (DC-SIGN) signaling pathway in GSEA analysis. Although CD209 has been reported to be low in placental chorionic villi [82], its activation has previously been associated with increased inflammatory signaling in endothelial cells [83] as well as increased inflammation and insulin resistance in adipocytes [84,85]. Additionally, the validated DEG involved in this pathway, *RPS6KA5*, encodes the mitogen-and-stress activated kinase 1 (MSK-1) protein that is highly expressed in placental trophoblasts [86,87]. Interestingly, MSK1 had previously been found to have an anti-inflammatory function via increasing the expression of the anti-inflammatory cytokine, IL-10 [88,89]. Overall, future studies are still needed to elucidate the global impacts of hyperglycemia on BeWo trophoblast inflammatory signaling pathways. Further, we observed an increased expression of LAP3 (involved in glutathione metabolism), a target that has previously found to be positively correlated with fasting blood glucose levels and has, by inhibiting autophagy, been implicated in the progression of Non-Alcoholic Fatty Liver Disease [90]. LAP3 has also been found to degrade glutathione (GSH), an important cellular antioxidant enzyme [91,92]. Overall, these data highlight an increased risk of oxidative stress development in hyperglycemia-exposed BeWo trophoblasts. As mitochondria are a predominant generator of Reactive Oxygen Species, a reduction in glutathione (GSH) availability may promote mitochondrial oxidative damage as well as increased mitochondrial fission, ultimately leading to mitochondrial dysfunction in high-glucose exposed trophoblasts, as reported to occur in other model systems [32,66,67].

### Metabolomic analysis of HG-cultured BeWo CT cells

Multivariate analysis of BeWo trophoblast metabolome profiles highlighted a divergence in metabolite signatures between HG and LG-cultured BeWo CT cells suggesting that high glucose levels also impact the levels of small, polar metabolites in the placenta. Subsequent pathway analysis highlighted specific intracellular accumulations of lactate (involved in the glycolysis/gluconeogenesis and pyruvate metabolism pathways), malonate (involved in the fatty acid biosynthesis pathway), as well as riboflavin (involved in the riboflavin metabolism pathway) in the HG-cultured BeWo CT cells.

The observed lactate accumulation likely suggests that glycolytic flux is in fact increased in HG-cultured BeWo CT cells, despite our concomitant observation of reduced HK2 expression [93]. It is interesting to note that this study did not observe increased basal or maximal glycolytic activity when assessed via the Seahorse XF Glycolysis Stress Test. As the Glycolysis Stress Test utilizes extracellular media acidification (resulting from the co-export of lactate and H^+^ from the cell) as a proxy measurement of glycolytic activity, this functional assay may underestimate glycolytic activity in the event of reduced lactate export as could potentially occur in a “Cytosol-to-Mitochondrial Lactate Shuttle” metabolic pathway [93].

Increased malonate levels may reflect an increase in *de novo* lipogenesis via FASN in HG-cultured BeWo CT and may be another mechanism underlying the increased TG levels observed in HG-cultured BeWo CT cells [94]. Future studies and the use of radio-labelled metabolites may be required to further characterize glycolytic activity and *de novo* lipogenesis in high-glucose exposed BeWo trophoblasts.

The accumulation of riboflavin (the essential vitamin B_2_) in HG-cultured BeWo CT cells either reflects an increased cellular uptake of riboflavin or an inhibition of riboflavin metabolism into the cofactors flavin adenine dinucleotide (FAD) and flavin mononucleotide (FMN) [95]. Reduced metabolism of riboflavin to its cofactor intermediaries has previously been implicated in the development of mitochondrial dysfunction [96]. However, riboflavin has also previously been suggested to act as an antioxidant [97] and has been demonstrated to be beneficial in reducing oxidative stress in rodent models of DM [98,99]. Future investigations may be needed to assess the impacts of riboflavin accumulation in HG-cultured trophoblasts and elucidate whether accumulation of this vitamin is beneficial or harmful to the placenta.

Interestingly, our integrated over-representation analysis combining DEGs and differentially abundant metabolites was not associated with an enrichment in any KEGG data sets with concomitant changes in both transcripts and metabolites. These data highlight limited overlap between the transcriptome and metabolome in high glucose treated trophoblasts and could indicate that BeWo trophoblast metabolism is primarily regulated by post-translational rather than transcriptional mechanisms. However, as BeWo placental trophoblasts are a male cell line, our data may also align with previous reports in human pregnancies that have found male placentae display relatively few adaptations to nutrient stresses *in vivo* compared to female placentae [82,83]. The presence and importance of these sex-dependent responses in placental trophoblasts likely highlights a limitation in the current study, and thus, the data reported here may only be predictive and representative of placental responses in pregnancies with a male fetus. Future studies are still needed to elucidate the differences in molecular mechanisms and responses between male and female placental trophoblasts in response to elevated glucose.

Finally, it is important to highlight that the choriocarcinoma origin of BeWo trophoblasts may limit the ability of our model culture system to fully represent *in vivo* placentation during hyperglycemia. While these data are a useful first outlook into the impacts of an isolated and prolonged *in vitro* hyperglycemic exposure on both progenitor CT cells, and differentiated SCT cells, future works utilizing primary-based placental material (including explant, as well as continuously improving trophoblast stem cell and organoid culture systems) are still needed to fully characterize villous trophoblast responses to high glucose.

## Conclusion

The results of the current study highlighted that a 72-hour hyperglycemia exposure independently impacts metabolic function and nutrient storage in BeWo trophoblasts, but does not mediate any global changes in functional readouts of mitochondrial respiratory activity. However, HG-cultured BeWo trophoblasts displayed markers suggestive of increased mitochondrial fission, as well as reduced antioxidant capacity, and we speculate this may highlight these HG-exposed cells are ultimately transitioning towards future mitochondrial dysfunction and failure. Notably, while the 72-hour hyperglycemic exposure utilized in the current study is relatively prolonged in the setting of *in vitro* cell culture experiments, this timeline is acute in comparison to the 40-week duration of human pregnancy *in vivo*. Thus, the data from the current study may highlight that preventing even short periods of hyperglycemia is of great importance in the clinical management of diabetic pregnancies. Maintaining appropriate glycemic management during gestation will help to ensure that placental mitochondrial function, and is sustained throughout gestation, and in turn limit the risk of the offspring developing later life non-communicable metabolic diseases.

## Supporting information

S1 File S1-S10 Figs. (PDF)

S2 File S1–S6 Tables. (XLSX)

S3 File Data used for statistical analysis and generation of figures. (XLSX)
