# Supplementary material for: The impact of hyperglycemia upon BeWo trophoblast cell metabolic function: A multi-OMICS and functional metabolic analysis
Source: PLoS One. 2023 Mar 17;18(3):e0283118. doi: 10.1371/journal.pone.0283118 (PMC10022812; doi:10.1371/journal.pone.0283118)
Supplement: S1 File — (PDF) [file pone.0283118.s001.pdf]

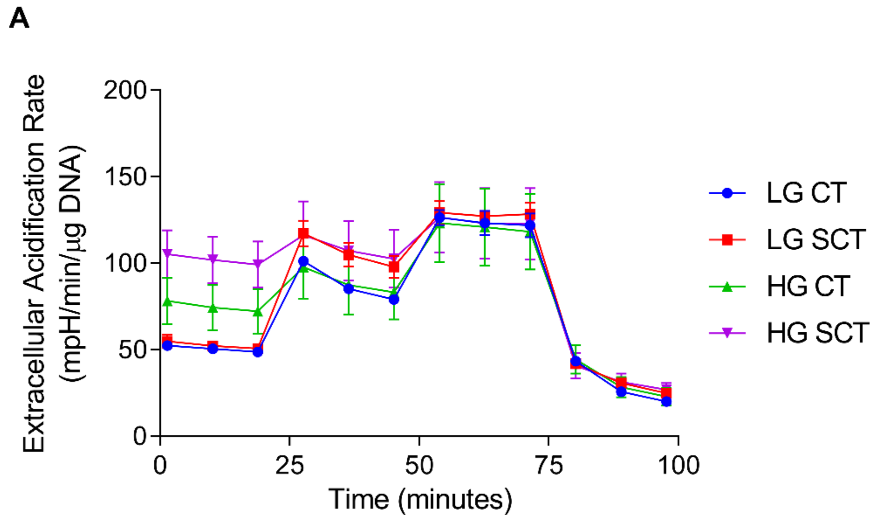

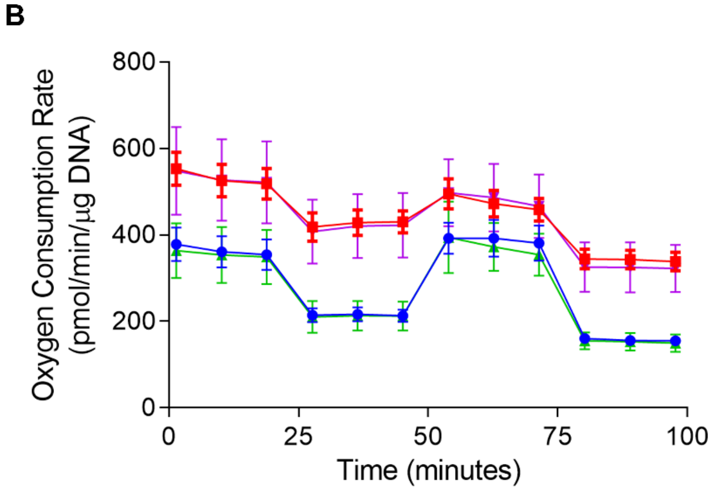

S1 Fig. Representative Seahorse Assay Tracings

(A) Representative Seahorse XF Glycolysis Stress Test assay tracings; data is presented as mean Extracellular Acidification Rate (ECAR) ± SEM of the technical replicates of each treatment group from one experiment. **(B)** Representative Seahorse XF Mito Stress Test assay tracings; data is presented as mean Oxygen Consumption Rate (OCR) ± SEM of the technical replicates of each treatment group from one experiment.

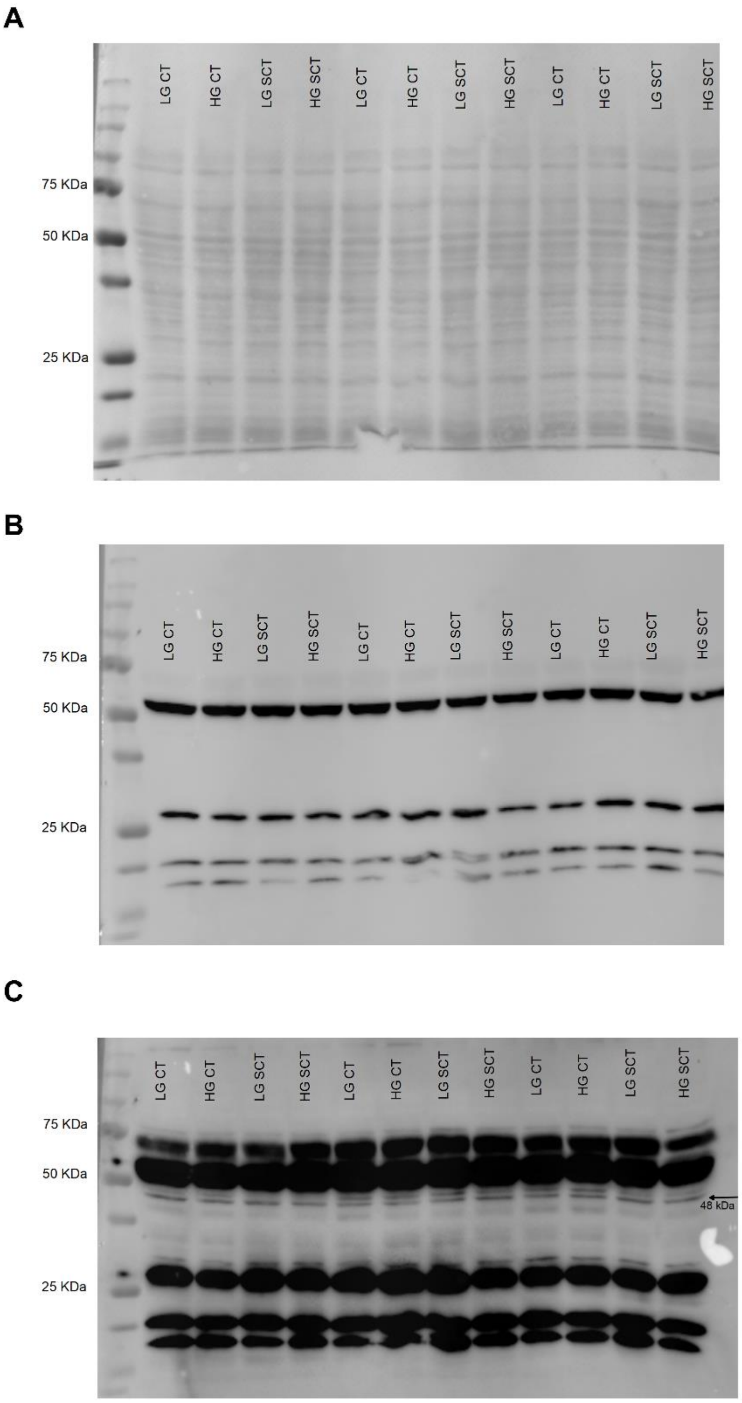

S2 Fig. Full length representative images of Mito Profile immunoblots.

(A) representative full-length ponceau image used to quantify total lane protein; **(B)** representative full length blot used to quantify band abundance for ETC complexes I (18 kDa), II (29 kDa), IV (22 kDa), and V (54 kDa) (9-second ChemiDoc exposure); **(C)** representative full length blot used to quantify band abundance for ETC complex III (48 kDa) (140-second ChemiDoc exposure).

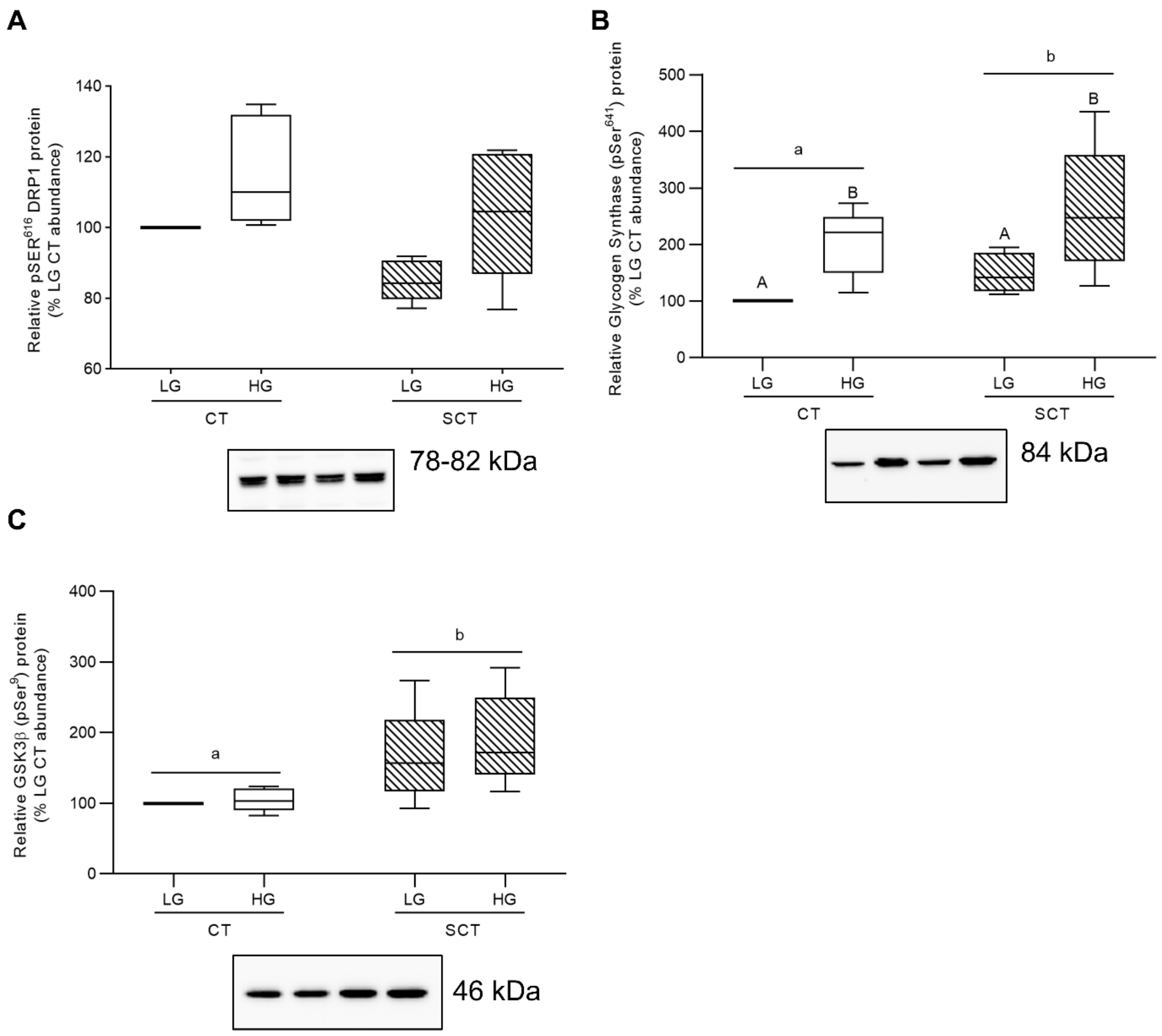

S3 Fig. Phosphorylated western blot target analysis relative to total lane protein.

Western blot analysis was performed to quantify the abundance of the post-translational modifications: **(A)** pSer^{616} DRP1, **(B)** pSER^{641} Glycogen Synthase, and **(C)** pSER^{9} GSK3β. Different lower-case letters denote differentiation state-dependent differences in protein abundance, and different upper-case letters denote glucose-level dependent differences within a differentiation state (Randomized-Block Two-Way ANOVA (2WA), and Sidak's multiple comparisons test, $p<0.05$, n=5/group). Protein band density was normalized to total lane protein (ponceau) for statistical analysis and the data is presented as percent of LG CT abundance for visualization (n=5/group).

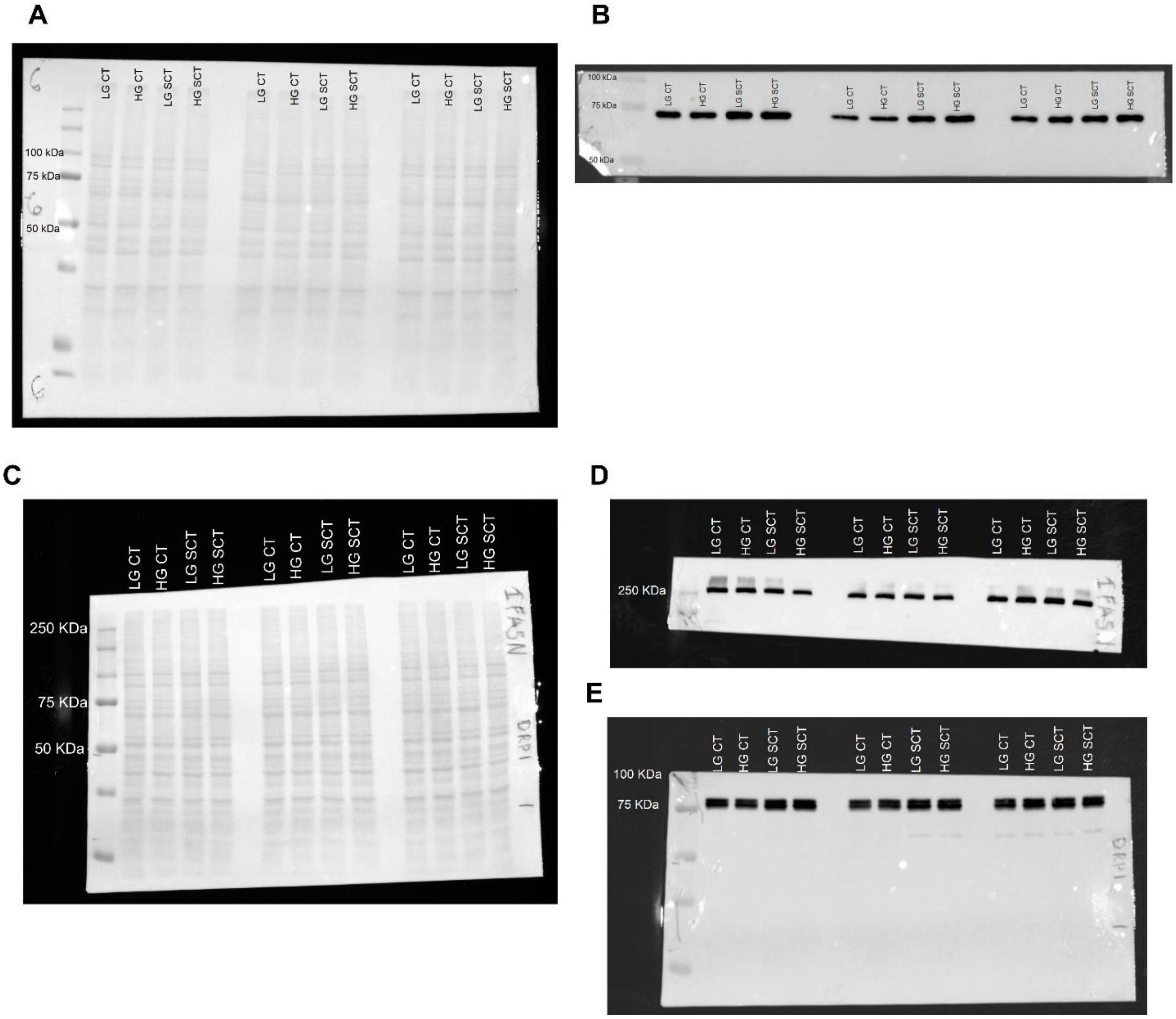

S4 Fig. Uncropped representative images of ACSL1, FASN, and total DRP1 immunoblots.

(A) Representative full length ponceau used to quantify total lane protein in ACSL1 immunoblots; **(B)** uncropped ACSL1 bands; **(C)** representative full length ponceau used to quantify total lane protein in FASN and total DRP1 immunoblots; **(D)** uncropped FASN bands; **(E)** uncropped total DRP1 bands.

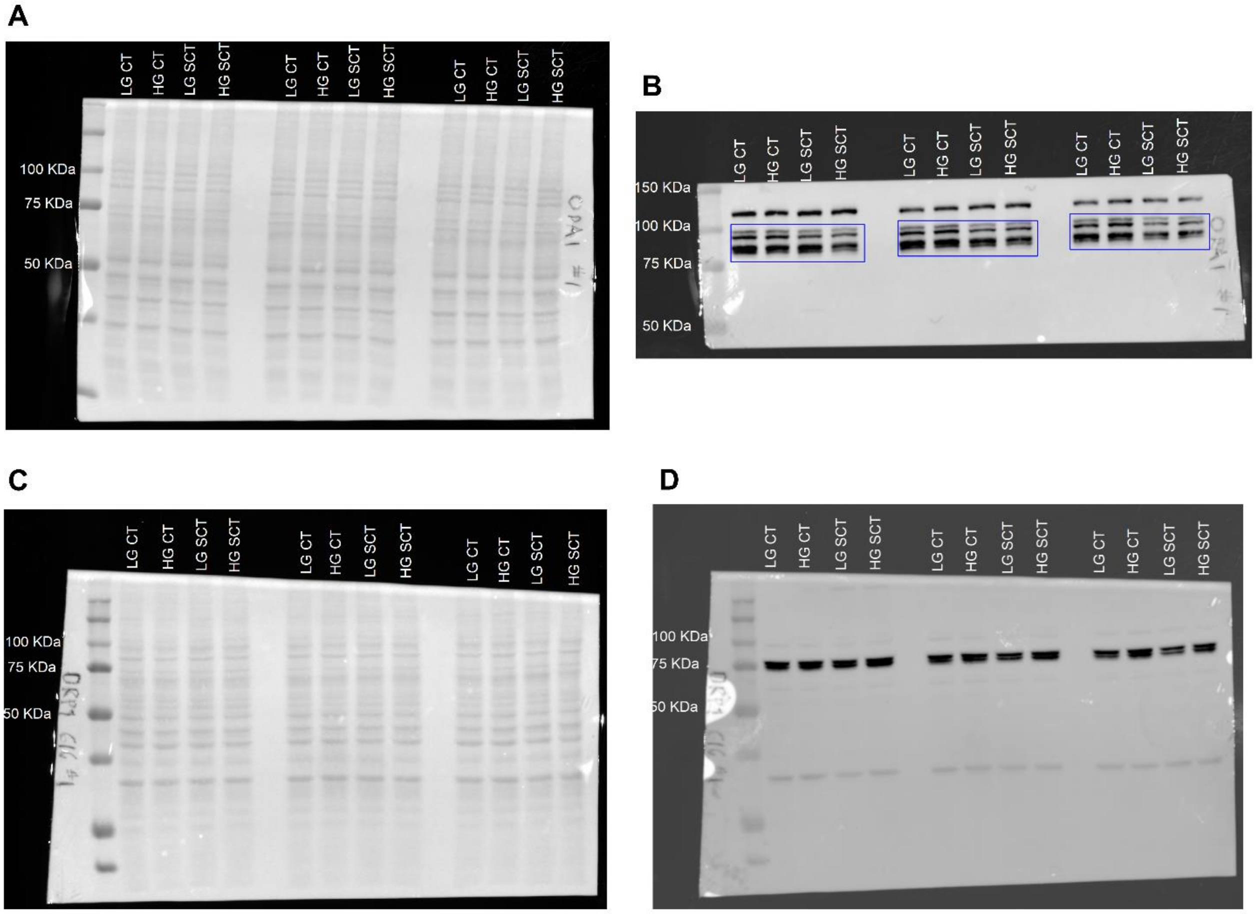

S5 Fig. Uncropped representative images of OPA1 immunoblots, and phosphorylated (Ser^{616}) DRP1.

(A) Representative full length ponceau used to quantify total lane protein in OPA1 immunoblots; **(B)** uncropped OPA1 bands; **(C)** representative full length ponceau used to quantify total lane protein in phosphorylated DRP1 immunoblots; and **(D)** uncropped pSER^{616} DRP1 bands. Bands between 80-100 KDa were utilized to quantify OPA1 protein abundance and are highlighted by blue boxes

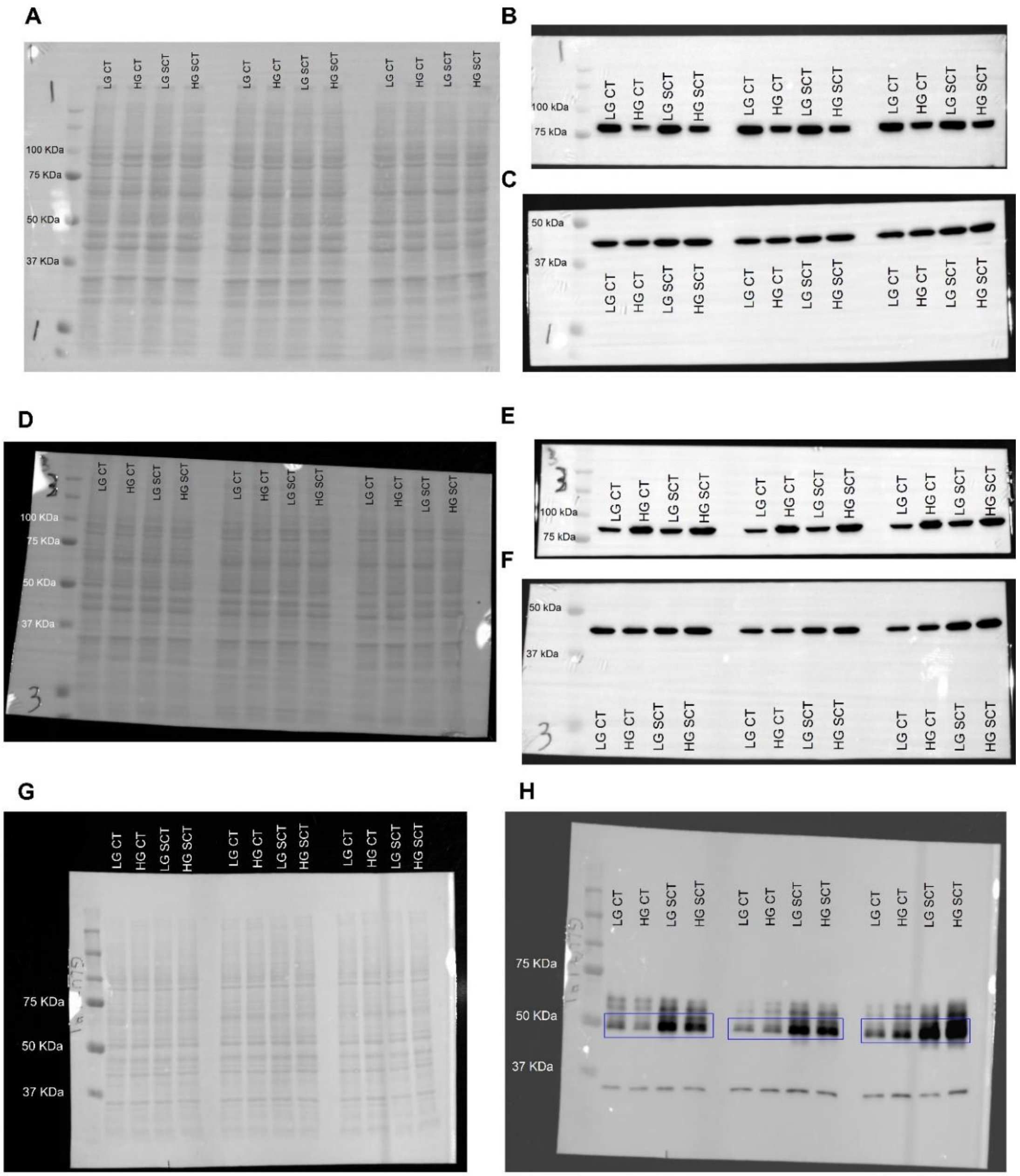

S6 Fig. Uncropped representative images of total and phosphorylated glycogen synthase and GSK3β immunoblots, as well as GLUT1 immunoblots.

(A) Representative full length ponceau used to quantify total lane protein in total glycogen synthase and GSK3β immunoblots; **(B)** uncropped total Glycogen synthase bands; **(C)** uncropped total GSK3β bands; **(D)** representative full length ponceau used to quantify total lane protein in phosphorylated glycogen synthase and GSK3β blots; **(E)** uncropped pSer^{641} Glycogen synthase bands; **(F)** uncropped pSer^{9} GSK3β bands; **(G)** representative full length ponceau used to quantify total lane protein in GLUT1 immunoblots; and **(H)** uncropped GLUT1 bands. Bands between 45-55 KDa were utilized to quantify GLUT1 protein abundance and are highlighted by blue boxes.

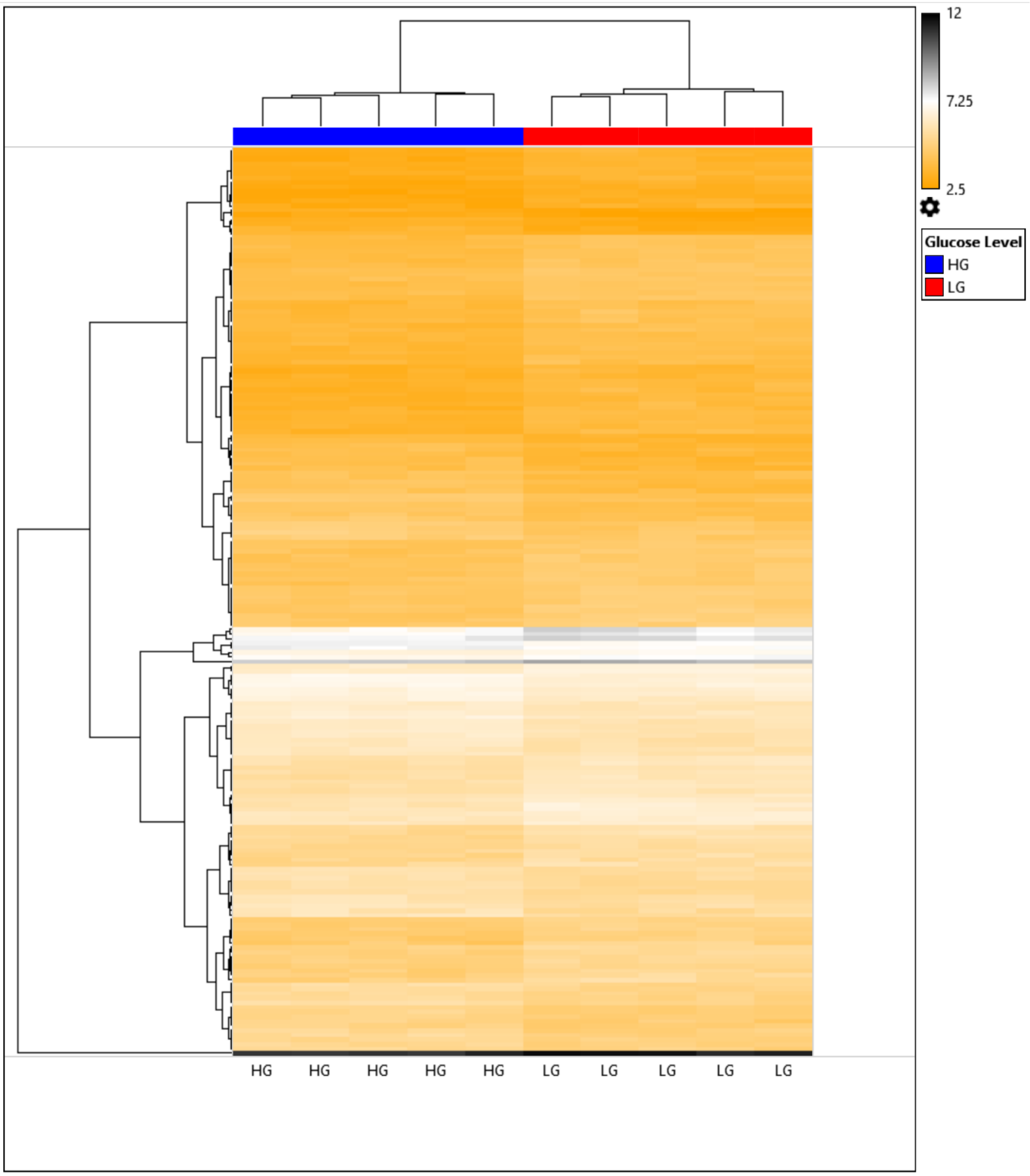

S7 Fig. Heat map visualization of differentially expressed genes in hyperglycemic (HG) cultured BeWo cytotrophoblast cells.

Each column of the heat map represents an individual sample, with blue columns representing HG-cultured BeWo CT cells and the red columns representing low-glucose (LG) cultured BeWo CT cells. Each row represents an individual differentially expressed gene. Gene expression intensity is color coded with orange representing low-expression genes and black representing high-expression genes.

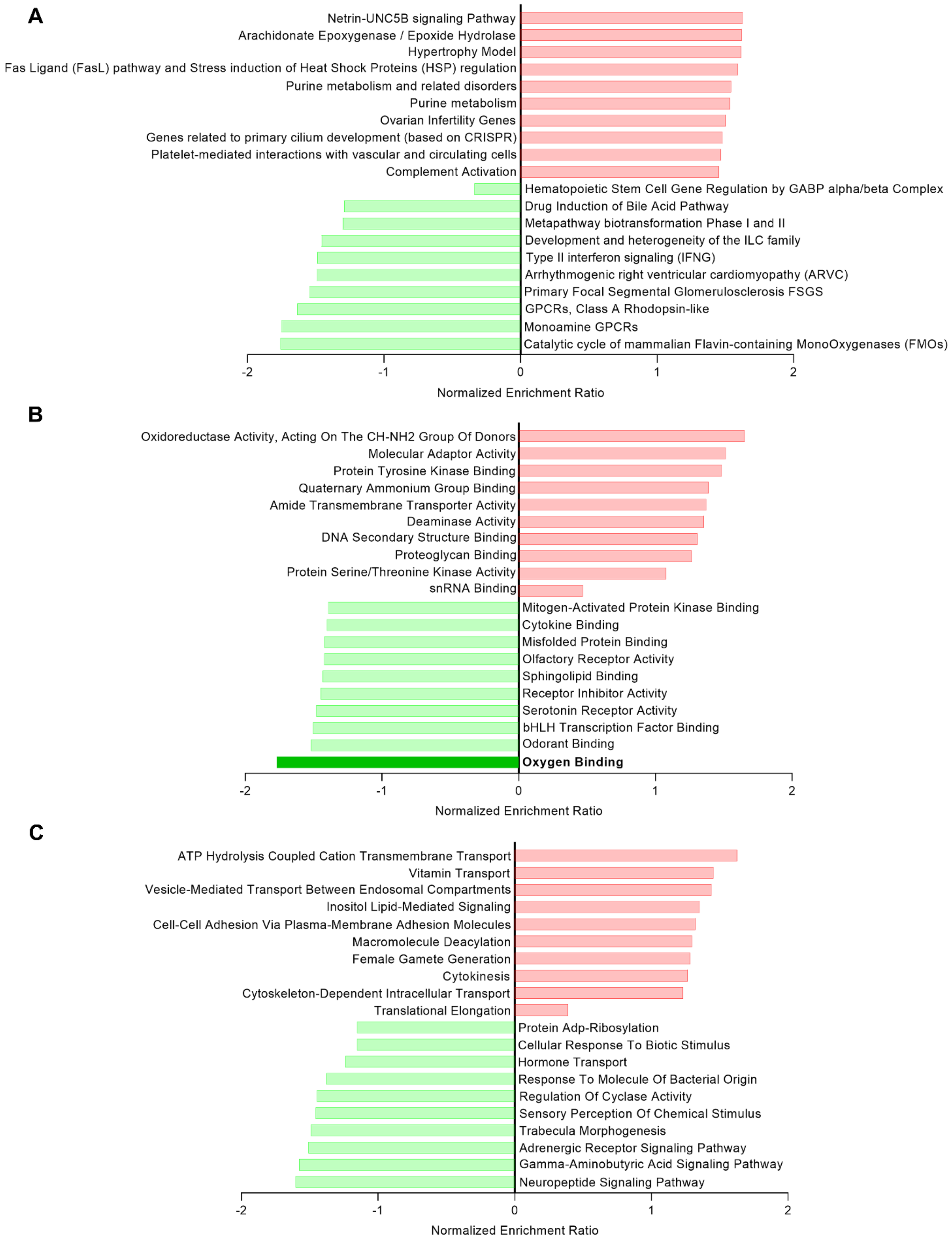
A
Netrin-UNC5B signaling Pathway
Arachidonate Epoxygenase / Epoxide Hydrolase
Hypertrophy Model
Fas Ligand (FasL) pathway and Stress induction of Heat Shock Proteins (HSP) regulation
Purine metabolism and related disorders
Purine metabolism
Ovarian Infertility Genes
Genes related to primary cilium development (based on CRISPR)
Platelet-mediated interactions with vascular and circulating cells
Complement Activation
Hematopoietic Stem Cell Gene Regulation by GABP alpha/beta Complex
Drug Induction of Bile Acid Pathway
Metapathway biotransformation Phase I and II
Development and heterogeneity of the ILC family
Type II interferon signaling (IFNG)
Arrhythmogenic right ventricular cardiomyopathy (ARVC)
Primary Focal Segmental Glomerulosclerosis FSGS
GPCRs, Class A Rhodopsin-like
Monoamine GPCRs
Catalytic cycle of mammalian Flavin-containing MonoOxygenases (FMOs)
-2
-1
0
1
2
Normalized Enrichment Ratio
B
Oxidoreductase Activity, Acting On The CH-NH2 Group Of Donors
Molecular Adaptor Activity
Protein Tyrosine Kinase Binding
Quaternary Ammonium Group Binding
Amide Transmembrane Transporter Activity
Deaminase Activity
DNA Secondary Structure Binding
Proteoglycan Binding
Protein Serine/Threonine Kinase Activity
snRNA Binding
Mitogen-Activated Protein Kinase Binding
Cytokine Binding
Misfolded Protein Binding
Olfactory Receptor Activity
Sphingolipid Binding
Receptor Inhibitor Activity
Serotonin Receptor Activity
bHLH Transcription Factor Binding
Odorant Binding
Oxygen Binding
-2
-1
0
1
2
Normalized Enrichment Ratio
C
ATP Hydrolysis Coupled Cation Transmembrane Transport
Vitamin Transport
Vesicle-Mediated Transport Between Endosomal Compartments
Inositol Lipid-Mediated Signaling
Cell-Cell Adhesion Via Plasma-Membrane Adhesion Molecules
Macromolecule Deacylation
Female Gamete Generation
Cytokinesis
Cytoskeleton-Dependent Intracellular Transport
Translational Elongation
Protein Adp-Ribosylation
Cellular Response To Biotic Stimulus
Hormone Transport
Response To Molecule Of Bacterial Origin
Regulation Of Cyclase Activity
Sensory Perception Of Chemical Stimulus
Trabecula Morphogenesis
Adrenergic Receptor Signaling Pathway
Gamma-Aminobutyric Acid Signaling Pathway
Neuropeptide Signaling Pathway
-2
-1
0
1
2
Normalized Enrichment Ratio

S8 Fig. Gene Set Enrichment Analysis of HG-cultured BeWo cytotrophoblast transcripts.

(A) Top 10 up-regulated and down-regulated Wikipathways gene sets and **(B)** top 10 up-regulated and down-regulated Gene Ontology (GO) molecular function gene sets, and **(C)** top 10 up-regulated and down-regulated GO biological processes gene sets (by normalized enrichment ratio) in HG-cultured BeWo CT cells. Green bars represent down-regulated gene sets and red-bars represent up-regulated gene sets, with dark green bars representing significantly down-regulated gene sets (FDR-p<0.25). The transcripts identified in the microarray were ranked by signal-to-noise ratio using the Gene Set Enrichment Analysis Software, and enrichment analysis was performed using WebGestalt.

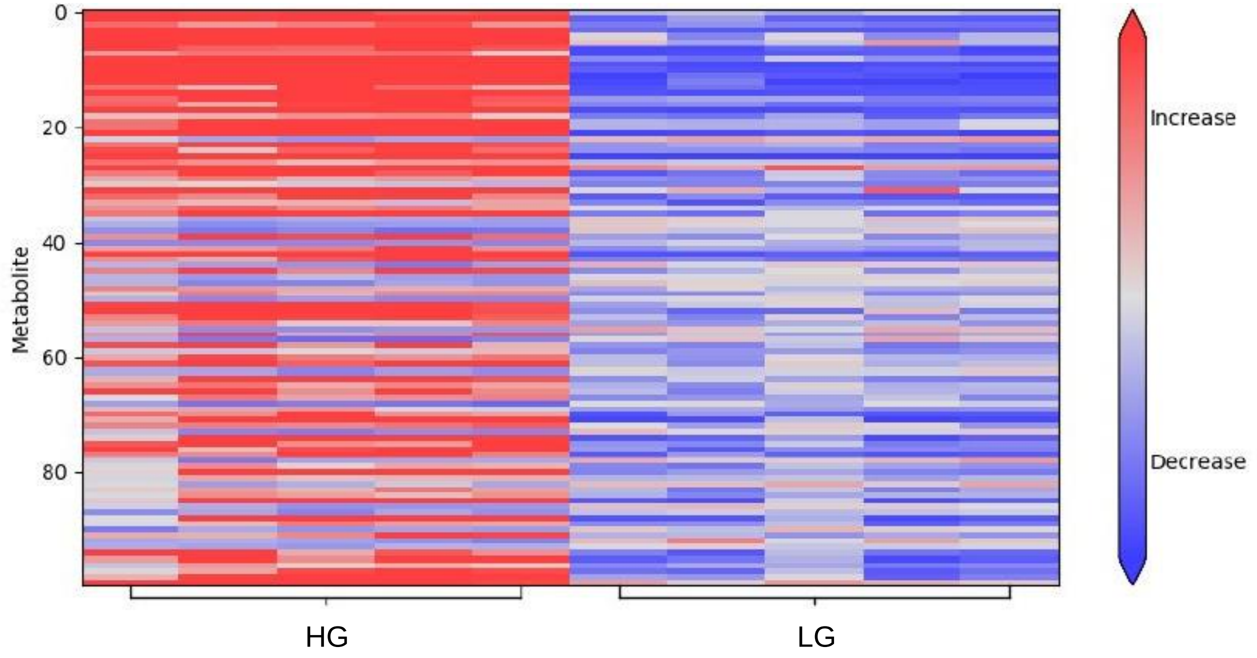

S9 Fig. Heap Map visualization of differentially abundant metabolites in hyperglycemic (HG) cultured BeWo cytotrophoblast cells.

A heat map was constructed to visualize the top-100 (by p-value) differentially abundant metabolites in HG-cultured BeWo CT cells. Each column represents a different sample, and each row represents an individual differentially abundant metabolite. Metabolite abundance was color coded with red representing metabolites with increased abundance and blue representing metabolites with decreased abundance.

A

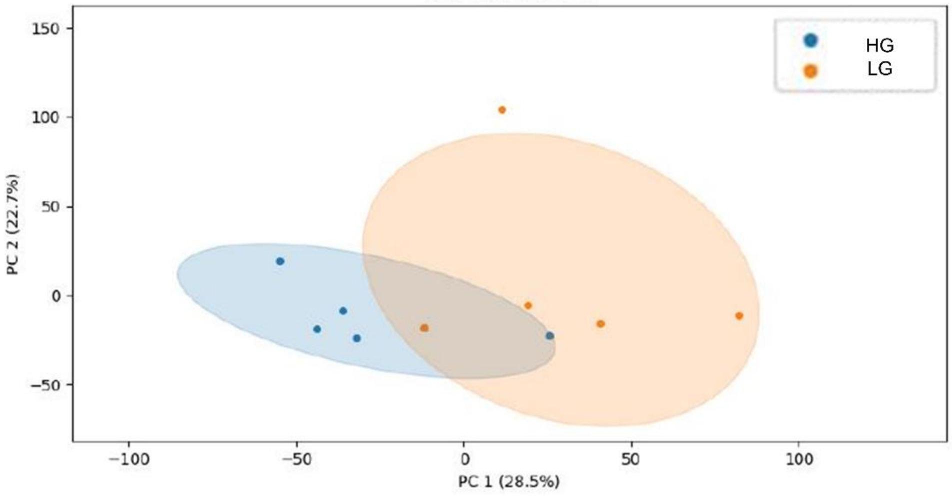

B

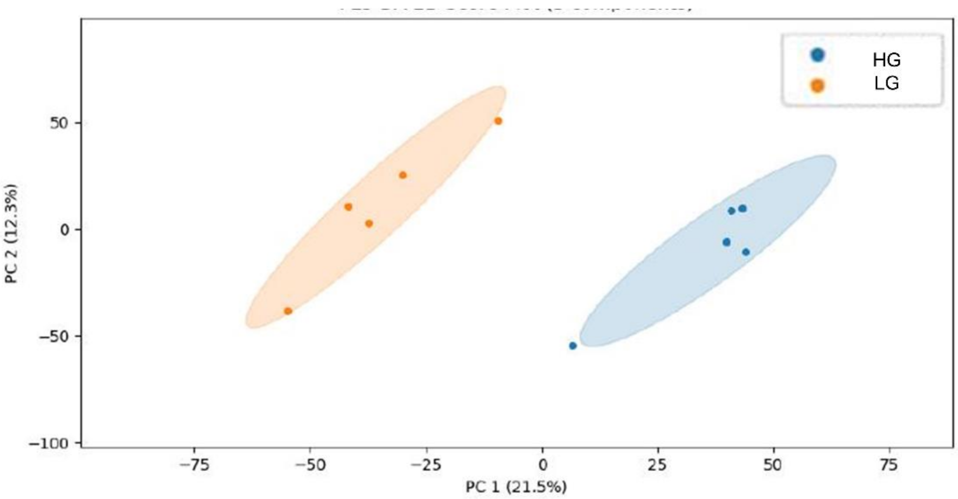

S10 Fig. Visualization of the degree of separation between metabolite profiles in HG and LG cultured BeWo CT cells.

(A) Unsupervised principal component analysis (PCA) and (B) supervised partial least squares discriminant analysis (PLS-DA) plots were constructed to visualize the separation in metabolite concentrations between LG and HG-cultured BeWo CT cells.
